# Repeated five-day administration of L-BMAA, microcystin-LR, or as mixture, in adult C57BL/6 mice - lack of adverse cognitive effects

**DOI:** 10.1038/s41598-018-20327-y

**Published:** 2018-02-02

**Authors:** Oddvar Myhre, Dag Marcus Eide, Synne Kleiven, Hans Christian Utkilen, Tim Hofer

**Affiliations:** 1 Department of Toxicology and Risk Assessment, Norwegian Institute of Public Health (NIPH), Oslo, Norway; 2 Department of Natural Sciences and Environmental Health, University College of Southeast Norway, Bø, Norway

## Abstract

The cyanobacterial toxins β-methylamino-L-alanine (L-BMAA) and microcystin-LR (MC-LR; a potent liver toxin) are suspected to cause neurological disorders. Adult male C57BL/6JOlaHsd mice aged approximately 11 months were subcutaneously injected for five consecutive days with L-BMAA and microcystin-LR alone, or as a mixture. A dose-range study determined a tolerable daily dose to be ~31 µg MC-LR/kg BW/day based on survival, serum liver status enzymes, and relative liver and kidney weight. Mice tolerating the first one-two doses also tolerated the subsequent three-four doses indicating adaptation. The LD_50_ was 43–50 μg MC-LR/kg BW. Long-term effects (up to 10 weeks) on spatial learning and memory performance was investigated using a Barnes maze, were mice were given 30 µg MC-LR/kg BW and/or 30 mg L-BMAA/kg BW either alone or in mixture for five consecutive days. Anxiety, general locomotor activity, willingness to explore, hippocampal and peri-postrhinal cortex dependent memory was investigated after eight weeks using Open field combined with Novel location/Novel object recognition tests. Toxin exposed animals did not perform worse than controls, and MC-LR exposed animals performed somewhat better during the first Barnes maze re-test session. MC-LR exposed mice rapidly lost up to ~5% body weight, but regained weight from day eight.

## Introduction

The cyanobacterial toxins β-methylamino-L-alanine (L-BMAA; non-endogenous *N*-alkylated amino acid) and microcystin-LR (MC-LR; ring-formed polypeptide) are suspected of causing developmental neurotoxicity and neurodegeneration in humans. Several cyanobacterial strains and diatoms produce L-BMAA^1^ and several cyanobacterial strains produce MC-LR which main function may be as a metal chelator in cyanobacteria^2,3^. Cyanobacterial toxins can be present in seafood, on vegetables, in drinking water etc., and L-BMAA and microcystins can co-occur^4^. Natural health products made of cyanobacteria (mainly produced by *Spirulina maxima* or *S*. *platensis* and *Aphanizomenon flos-aquae*) are of particular concern as they may contain both L-BMAA and MC-LR^5^, and contamination from co-growing *Microcystis* (produce microcystins) can occur^6^.

L-BMAA has long been a suspected cause of the increased incidence of amylotrophical lateral sclerosis-Parkinson’s dementia complex (ALS-PDC, or Lytico-bodig disease), a slowly progressing disease, affecting the Chamorro people of Guam in the mid-20^th^ century. During World War II, the Guamanians were undernourished and their diet consisted of cycad seed flour containing L-BMAA (roots of cycads live in symbiosis with cyanobacteria) as well as flying foxes and other animals that consume cycad seeds (L-BMAA is biomagnified in the food chain)^7^. For development of PDC, the critical age of exposure (unknown agent) appears to have been during adolescence and adulthood^8^, whereas exposure at all ages can have led to ALS^9^ which may be triggered at higher doses. Individual genetic susceptibility, nutrient intake and co-exposures from other toxicants may have influenced disease onset. The latency period may have lasted up to decades in some cases^8,9^. L-BMAA is not acutely toxic; in young Swiss mice, the presumptive LD_50_ by intraperitoneal injection (i.p.) is 300 g/kg body weight (BW)^10^. The plasma elimination half-life is 1.7 days in adult C57BL/6 mice^11^ and ∼1 day in adult rats^12^. L-BMAA can act upon excitatory amino acid (e.g. glutamate) receptors as an agonist in presence of bicarbonate^13^, causing excitotoxicity and neuronal damage, but can also be incorporated into proteins^11^ (total L-BMAA levels are therefore difficult to measure, requiring acid hydrolysis of proteins^5^), replacing L-serine and causing protein misfolding^14^. Brain uptake of L-BMAA is less than 1%, but once taken up, L-BMAA persists in the brain for days or even weeks^11^. L-BMAA has been thought to mainly affect neurons in motor area regions which is supported by two high-dose long-term administration studies in adult monkeys^15^ and rats^16^. However, some high-dose long-term studies on adult rodents^17,18^ and adult monkeys^19^ did not observe motor related effects. Motor related effects are reported to disappear after halted administration^20,21^. Thus, long-term progressive ‘ALS-PDC like effects’ have been difficult to reproduce in animals^7^. Interestingly, effects on spatial learning/memory were observed in adult rodents after subcutaneous (s.c.) high-dose L-BMAA administration in young pups^22,23^, but few studies have yet investigated learning/memory after administration in adult rodents. L-BMAA passes the blood-brain barrier (BBB) possibly through two-three different mechanisms, enters several brain compartments^11^, and particularly damages the mouse hippocampus at high dose^24^. Brain uptake was somewhat higher in male than female young Swiss mice^10^.

MC-LR is reported to be developmental neurotoxic in young rats and both single^25^ and long-term (14–56 days) repeated low-dose administration affected spatial learning and memory^26–28^. However, no behavioral studies after MC-LR administration in adult rats or in mice has yet been performed. MC-LR is taken up into cells by organic anion-transporting polypeptide (OATP) active transporters present also in the BBB^29,30^. MC-LR enters the target organ liver through bile import, and within cells, MC-LR has strong affinity (can form covalent bonds) to serine/threonine phosphates and acts as an inhibitor of this enzyme group^31^. Through this interaction, a cascade of possible events for cytotoxicity and genotoxicity is initiated^31^. Particularly, ubiquitously expressed Ser/Thr-specific protein phosphatase PP1 and PP2A^32^ are targets that are most potently inhibited by MC-LR^33^, resulting in hyperphosphorylation of proteins disturbing cellular signaling and causing cytoskeletal (microtubule) integrity issues^34^. Typically, animals die from intrahepatic hemorrhage within hours. The plasma half-life of MC-LR is only minutes^35^, whereas covalently bond MC-LR inside the liver was found to persist for six^35^ and 14 days^36^, respectively. Reported single acute LD_50_ doses for MC-LR in mice vary upon administration route, strain and age (three-week-old mice were more vulnerable than one- or two-week-old)^37^ and survival curves are often very steep, implying that a very small dose increase can drastically increase mortality. Two single dose studies in adult (8–12-week-old, ~25 g) Swiss albino mice of the Hale-Stoner strain reported LD_50_ to be 36 µg/kg BW (i.p. in males and females)^38^, and 60 µg/kg BW (intravenous (i.v.) or i.p. in females)^39^, respectively. For Balb/c mice, LD_50_ was 32.5 µg/kg (i.p. in males at various ages weighing 20–35 g)^40^, and 65.4 µg/kg (i.p.) and 10.9 mg/kg (oral) in 6-week-old females^41^. The liver and kidneys are damaged initially, but then undergoing reparative processes in surviving animals that are clinically affected (raised levels of serum liver enzymes, display of weakness, etc.). Pretreatment with the cyclic peptide cyclosporine A^36,42–46^ and rifampin^36,45–47^, both broad OATP inhibitors^48^, effectively protects from MC-LR’s acute liver toxicity. Also sub-lethal doses of MC-LR can offer self-protection if administrated hours to days prior to normally lethal doses of MC-LR^40,45,46^.

**Figure 1:**
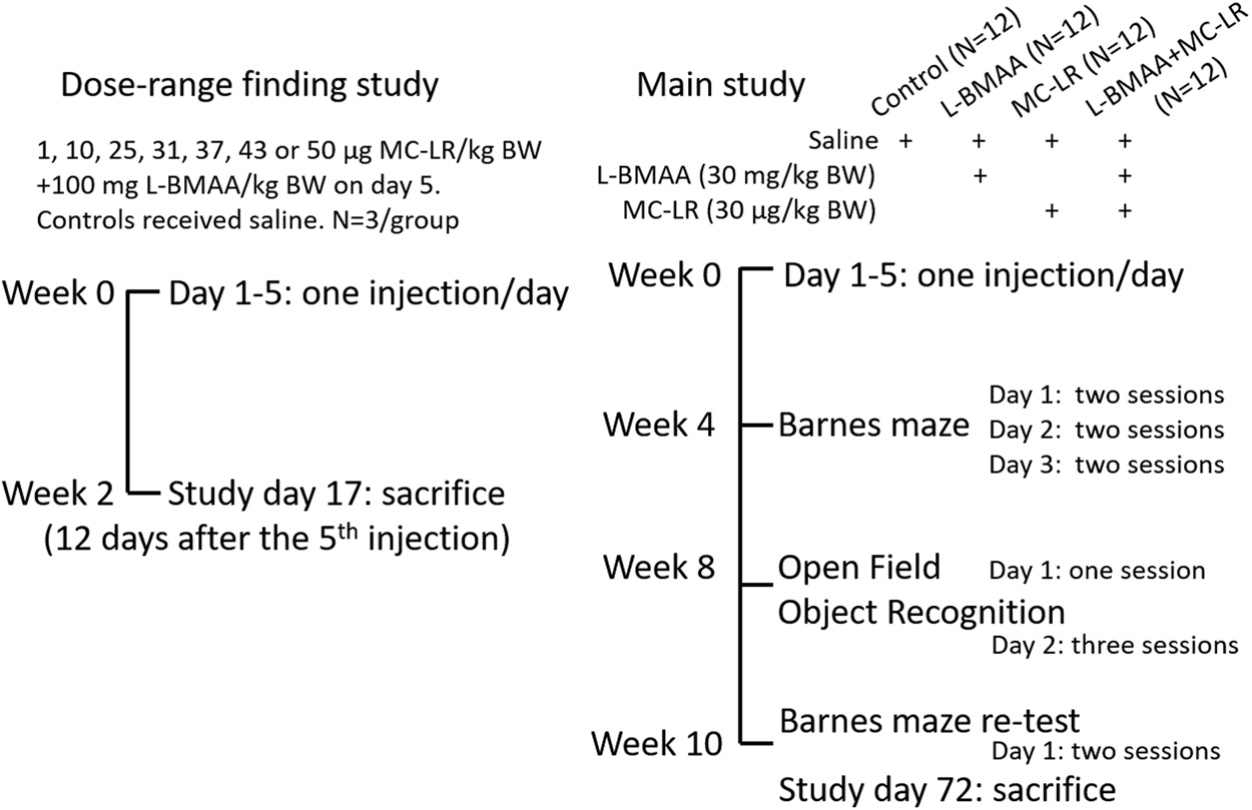
Study designs for the dose-range finding and main studies. The dose-range finding study was performed to determine a suitable tolerable dose of MC-LR to be used in the main study, which investigated long-term behavioral effects.

In this study we were interested in if five-day repeated daily administrations of L-BMAA and MC-LR alone, or in combination, in adult 11-month-old (male C57BL/6 become about 26 months) mice would cause long-term behavioral effects. Since MC-LR is a potent liver toxin, a five-day repeated dose-range finding study was first performed to determine a tolerable dose (Fig. 1). For the first time, this study investigates the effects of MC-LR on behavior (learning and memory, spontaneous activity and anxiety) in adult animals, and the combinatorial effect of MC-LR + L-BMAA. In the Barnes maze, seven parameters were quantified: startdirection error (novel parameter), primary latency, escape latency, number of primary errors, number of total errors, mean velocity and distance travelled. The effect of scopolamine (an anticholinergic drug)^49^ administration (positive control) immediately prior to Barnes maze testing was investigated using a separate set of mice to check for which parameters that get affected during impaired spatial learning and memory. The chosen five-day exposure period is supposedly more suitable than a single dose as mice could die, and could for instance resemble adult exposure from a batch of contaminated food consumed over a few days (e.g. vegetables and/or seafood), a visit to a summerhouse over a few days where the drinking and/or swimming water is contaminated (lake activities and wind can result in aerosol/droplet formation), or consuming a contaminated batch of cyanobacterial food supplements for a few days.

## Results

### Dose-range finding study – MC-LR tolerability

**Figure 2:**
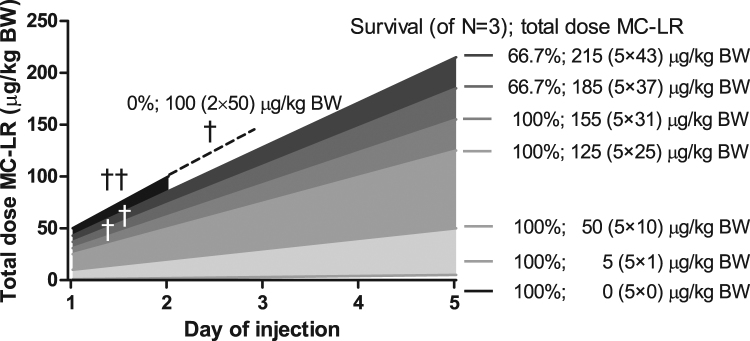
Survival and tolerated MC-LR doses by mice in the five-day repeated dose-range finding study. On the fifth injection, all mice except controls were co-administrated 100 mg L-BMAA/kg BW which is not acutely toxic. Time-points of deaths are indicated with a dagger (†).

**Figure 3:**
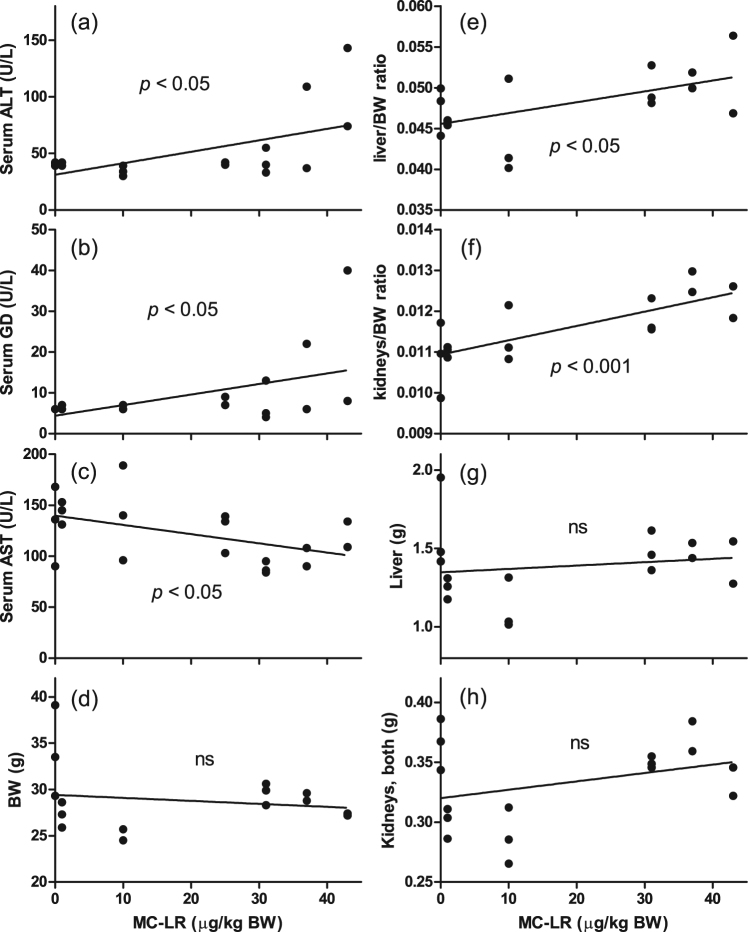
MC-LR related effects on mice serum parameters, organ and body weights in the dose-range finding study. (**a**) Serum ALT (U/L), (**b**) serum GD (U/L), (**c**) serum AST (U/L), (**d**) BW (**g**), (**e**) liver/BW, (**f**) kidneys/BW, (**g**) liver (**g**), and (**h**) kidneys (both, **g**) weight at sacrifice (day 17). Slopes that significantly deviate from zero using linear regression are indicated with p-values whereas ‘ns’ means not significantly different from zero. Body and organ weights were not recorded in the 25 μg/kg BW group due to an error. The unexposed control group contained one abnormally large mouse.

MC-LR is known to be a potent liver toxin with a steep acute dose-response relationship wherefore an initial tolerability study was performed. The toxic effect from repeated one-week (five days) MC-LR administration has previously not yet been reported, but published literature on acute adverse toxicity (LD_50_) for single administration provided some guidance. Thus, initially we evaluated the effect of repeated (five-day) MC-LR administration at 1 and 10 μg/kg BW. However, at these dose levels, no apparent toxicity was observed. Therefore, a modest 2.5- and 5-fold increase from 10 μg/kg BW was tested. All mice survived at 25 μg/kg BW displaying no apparent clinical toxicity, but at 50 μg/kg BW all three mice accidentally died (one mouse died after the second injection so it is unclear if it would have survived the first injection alone), Fig. 2. To further explore the maximum tolerable MC-LR dose to be used in the main study, the doses 31, 37 and 43 were tested along with the 0 (control) μg/kg BW group. No apparent clinical toxicity was observed at 31 μg/kg BW, but at 37 and 43 μg MC-LR/kg BW one out of three mice died within 24 h after the first injection (Fig. 2). Interestingly, the mice (two out of three) surviving the first 37 and 43 μg/kg BW doses, as well as all mice given lower (1–31 μg/kg BW) doses, tolerated all five doses; thus, in total up to 215 μg MC-LR/kg BW. Moreover, these mice displayed no apparent motor related effects or other issues when observed in cages or during handling up to sacrifice on day 17 (12 days after the fifth injection). Co-administration with L-BMAA on day five had no observable effect. Repeated injections at doses ≥ 25 μg/kg BW caused slight BW reductions (at the most ~5%) that lasted from injection day three to study day 12 after which these survivors regained weights (Supplementary Table S1 and Supplementary Fig. S1). However, also the controls displayed slight (3–4%) BW reductions for unknown reasons. The 1 and 10 μg/kg BW groups, however, displayed no BW reductions. The acute LD_50_ for a single injection could not be precisely determined due to a low number of mice tested but lay in the range between 43 (33.3% dead) – 50 (66.7–100% dead) μg MC-LR/kg BW. For five consecutive daily injections, a tolerable dose was determined to be ~31 μg MC-LR/kg BW based on 100% survival (Fig. 2) and the analyses (organ/BW ratios, serum liver enzymes) at sacrifice, Fig. 3.

### Dose-range finding study – body and organ weights

At sacrifice (day 17), the trend lines for liver/BW and kidneys/BW ratios significantly increased with the dose of MC-LR, whereas for body, liver and kidneys (both) weights the trend lines did not significantly differ from zero, Fig. 3. Data are available in Supplementary Table S2.

### Dose-range finding study – liver enzymes in serum

At sacrifice (day 17), the trend lines significantly increased with the dose of MC-LR for serum ALT and GD levels, whereas the trend line significantly decreased for AST, Fig. 3. Data are available in Supplementary Table S3.

### Main study – body and organ weights, and clinical observations

**Figure 4:**
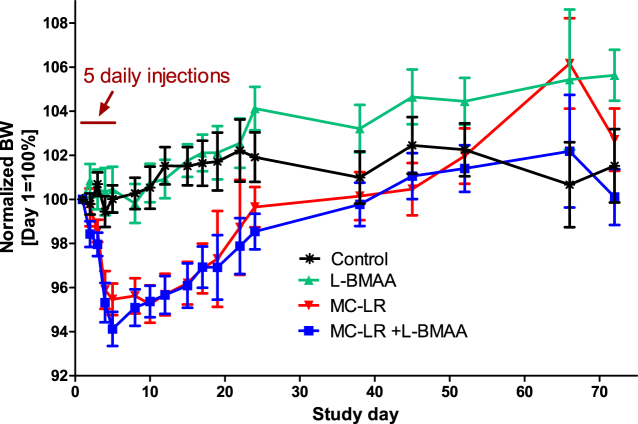
Normalized BWs (%) for the four groups of mice in the main study over 72 days. Data shown as mean ± SEM (N = 12/group and time-point except on days 19, 22 and 66 when BWs for only one of the two subsets were recorded (N = 6/group)).

Mice receiving repeated (five-day) 30 μg MC-LR/kg BW administration immediate begun to lose weight, Fig. 4 (Supplementary Table S4). On day five, BW reductions were on average 5.2% compared to day one for the two groups receiving MC-LR, whereas the L-BMAA and control group did not display any BW reduction. BWs were not recorded on day six or seven, but from day eight and onwards the MC-LR exposed mice regained BW. No indications of abnormal behavior or clinical toxicities (e.g. motor function) was observed for any of the groups during the 72 day study period. Overall, the average BWs for all four groups increased slightly (2.6%) from 29.44 g (day one) to 30.20 g (day 72). At sacrifice (day 72), liver or kidneys (both) weights, liver/BW and kidneys/BW ratios did not significantly differ among the four groups (one-way ANOVA analyses) (Supplementary Table S5).

### Main study – behavioral testing

#### Barnes maze

**Figure 5:**
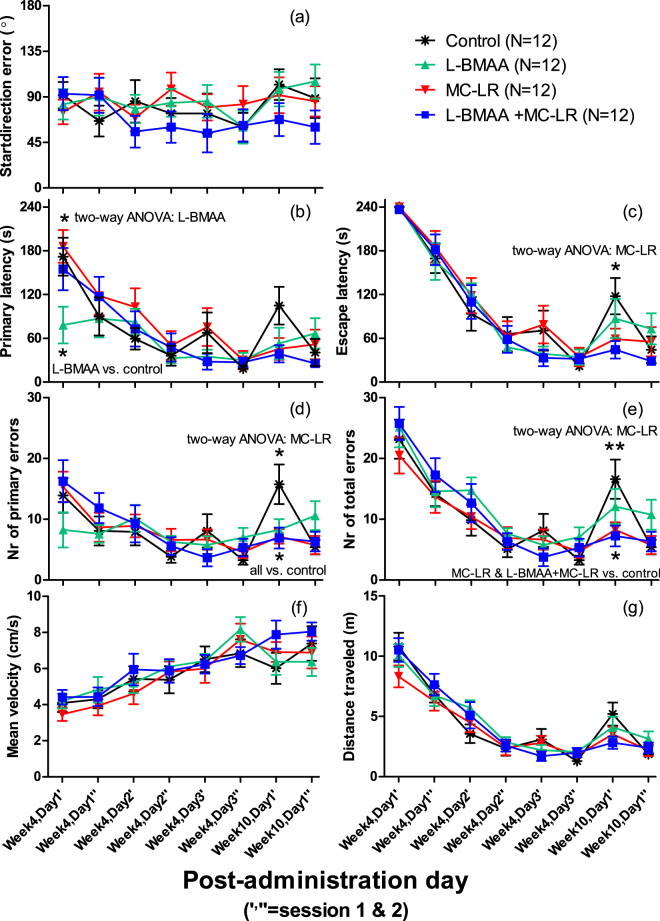
Behavioral performance of mice in the Barnes maze over eight consecutive sessions. (**a**) Startdirection error (0 to 180 degrees), (**b**) primary latency (s), (**c**) escape latency (s), (**d**) number of primary errors, (**e**) number of total errors, (**f**) mean velocity (cm/s), and (**g**) distance travelled (m). Testing was performed four (over three consecutive days) and 10 (on one day) weeks after administration with two sessions (1 = morning; 2 = afternoon) per day. Data are shown as mean ± SEM. Significant main effects by two-way ANOVA and significant differences among the groups by Dunnett’s t-test at the respective session are indicated with asterisks (*p < 0.05 and **p < 0.01) above and below the curves, respectively.

No adverse behavioral anomalies due to the cyanobacterial toxins, either alone or in combination, were observed vs the controls in the Barnes maze and no behavioral changes were observed for animals in their cages or when handled during weight recordings or else during the whole 72 day study period. For the seven parameters (Fig. 5; data are available in Supplementary Tables S6–S12), no significant effects were observed when comparing the exposed groups’ respective curves (or sections of the curves) against the control group using repeated measures ANOVA. For startdirection error, a slight non-significant progressive improvement (slope −4.2°/session) was observed during the first three days (six sessions) for the control group, but which was lost during the re-test sessions (Fig. 5a). However, the L-BMAA exposed groups displayed a significantly (*p* = 0.020) shorter Primary latency than non-L-BMAA exposed groups at the first session (Fig. 5b) using two-way ANOVA. In agreement, the L-BMAA treated group displayed significantly shorter primary latency than the control group (*p* = 0.036) at this first session with Dunnett’s *t*-test. As this was the very first encounter with the Barnes maze, it is unclear why L-BMAA exposed animals would find the correct hole faster than the control group (the L-BMAA + MC-LR group had similar primary latency as the control group). The two-way ANOVA identified that MC-LR exposed groups performed significantly better than the non-MC-LR exposed groups at the first re-test (seventh) session for three different Barnes maze parameters: i) the MC-LR exposed groups displayed a significantly (*p* = 0.018) shorter escape latency than non-MC-LR exposed groups (Fig. 5c). ii) Likewise, the MC-LR exposed groups had a significantly (*p* = 0.025) lower number of primary errors than non-MC-LR exposed groups (Fig. 5d). In agreement, all three exposed groups had a significantly lower number of primary errors compared to the control group using Dunnett’s *t*-test (L-BMAA vs. control: *p* = 0.044; MC-LR vs. control: *p* = 0.018; L-BMAA + MC-LR vs. control: *p* = 0.015). iii). Moreover, the MC-LR exposed groups had a significantly (*p* = 0.009) lower number of total errors than non-MC-LR exposed groups (Fig. 5e). The MC-LR and L-BMAA + MC-LR groups had a significantly lower number of total errors compared to the control group using Dunnett’s *t*-test (*p* = 0.049 and 0.024, respectively). Notably, the control group for some reason contained some animals with poor recollection at the first re-test (seventh) session.

#### Open Field (OF), Novel Location Recognition (NLR) or Novel Object Recognition (NOR)

**Figure 6:**
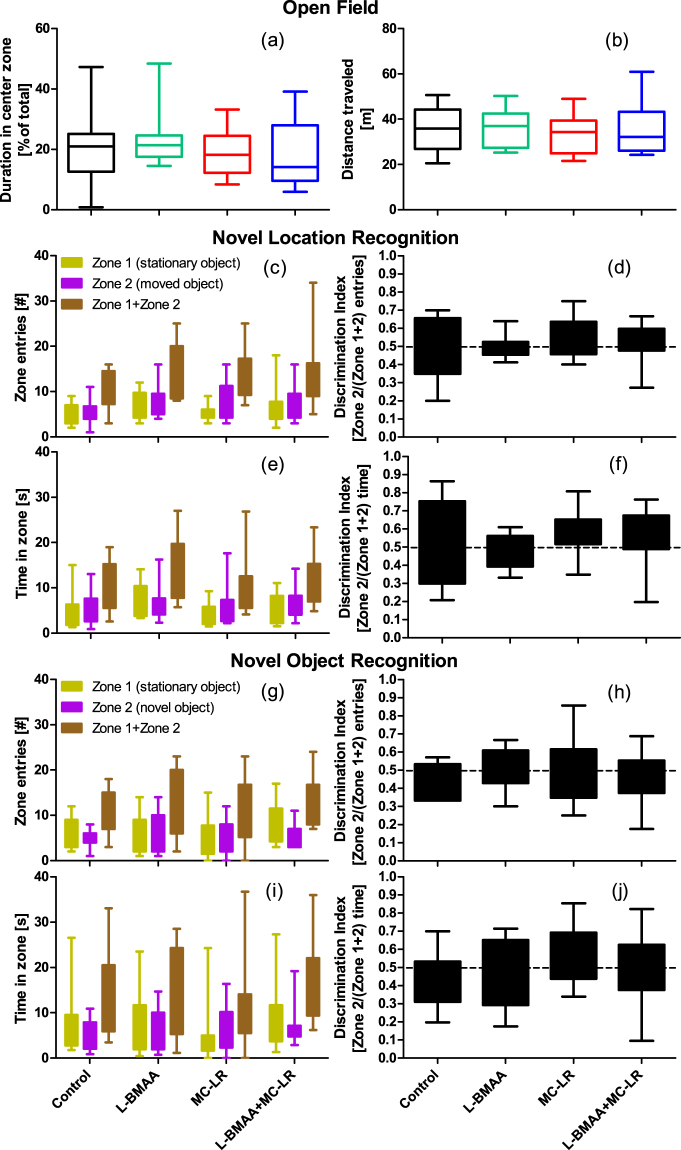
OF, NLR and NOR tests of mice during week eight. On the first day, OF tests were performed during 10 min with recording of (**a**) the time spent (shown as % of total) in the center (25 × 25 cm) zone inside white squared (50 × 50 cm) boxes, and (**b**) total distance travelled (m). The following day begun with a 5 min acclimatization period where two identical objects had been placed inside the box. Thereafter the NLR (**c–f**) and NOR (**g–j**) tests were performed (5 min each) on the same day with 2 h in-between the sessions. Number of entries (#) and time (s) spent in the zones surrounding the two objects were calculated from which discrimination indexes were calculated. N = 12 in all groups. Data are shown as mean ± SEM.

No significant treatment related effects were found for OF, NLR and NOR, Fig. 6 (data are available in Supplementary Tables S13–S15). For NLR and NOR, a discrimination index (is 0.5 if no difference in time spent (or entries into zones) exploring the changed or the unchanged object) was calculated as previously described^50^.

#### Effect of scopolamine on the seven quantified Barnes maze parameters

**Figure 7:**
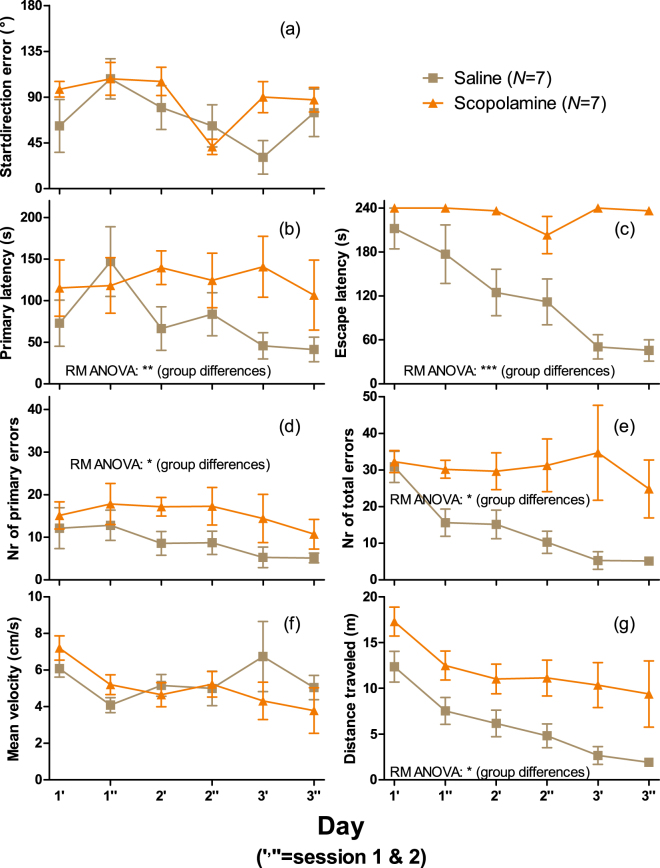
Positive control test using scopolamine in the Barnes maze to assess spatial learning and memory performance in mice by the seven quantified parameters. (**a**) Startdirection error (0 to 180 degrees), (**b**) primary latency (s), (**c**) escape latency (s), (**d**) number of primary errors, (**e**) number of total errors, (**f**) mean velocity (cm/s), and (**g**) distance travelled (m). Testing was performed 15 min after saline or scopolamine i.p. administration (1 mg/kg BW) over three consecutive days with two sessions (1 = morning; 2 = afternoon) per day. Data are shown as mean ± SEM. Significant differences between the two groups (1 mg/kg BW scopolamine or saline injected i.p. 15 min prior to each session) by repeated measures (RM) ANOVA are indicated with asterisks (*p < 0.05, **p < 0.01, and ***p < 0.001).

Scopolamine strongly disturbed the spatial learning and memory performance during repeated Barnes maze testing over several training sessions, Fig. 7 (data are available in Supplementary Tables S16–S22). The following results were obtained when comparing scopolamine treated vs. control data (curves) using repeated measures ANOVA (other tests were not performed): (a) startdirection error: no difference between the two group’s overall means, curve shapes are different (*p* = 0.026 for time*group interaction), a slight non-significant progressive improvement (slope −4.9°/session) for the controls but no effect for both groups together; (b) primary latency: difference between the groups (*p* = 0.0099), curve shapes are not different, no progressive improvement for the controls (and also not for both groups together); (c) escape latency: difference between the groups (*p* < 0.001), curve shapes are different (*p* < 0.001), progressive improvement for the controls (*p* = 0.047); (d) number of primary errors: difference between the groups (*p* = 0.014), curve shapes are not different, no progressive improvement for the controls (and also not for both groups together); (e) number of total errors: difference between the groups (*p* = 0.022), curve shapes are not different, progressive improvement for the controls (*p* = 0.032); (f) mean velocity: no difference between the groups, curve shapes are not different, no progressive decrease for the controls alone, but for both groups together (*p* = 0.019); (g) distance travelled: difference between the groups (*p* = 0.018), curve shapes are not different, a progressive decrease for the controls (*p* = 0.0026).

## Discussion

Controversy surrounds the proposed hypothesis that L-BMAA or MC-LR exposure through consumption of cyanobacteria (or their toxins) contaminated health supplements, foods (e.g. fish, shellfish and vegetables) or drinking water could play a causal role in various neurodegenerative pathological conditions^51^. It is well known that early-life exposure to the cyanobacterial toxins L-BMAA and MC-LR can cause developmental neurotoxicity in animal models^23,28^. However, since there is a knowledge gap on post-pubertal exposure and neurotoxic effects in adult animals, we investigated spatial learning and memory performance of these cyanobacterial toxins (separate and combined) in roughly one-year-old mice. This exposure regime is of relevance for suspected neurological diseases like PDC, since the critical age of exposure to L-BMAA appears to have been during adolescence and adulthood^8^, whereas exposure at all ages could be a risk factor for ALS^9^. In our study, significant treatment-related adverse effects from toxin exposure (MC-LR, L-BMAA or MC-LR + L-BMAA) were not observed for any parameters in any of the behavioral tests performed, Figs 5 and 6, or during the whole study period over 10 weeks. Surprisingly, MC-LR exposed animals had a somewhat better long-term spatial memory at the first Barnes maze re-test (week 10) than non-MC-LR exposed animals, Fig. 5. It is unclear why, but initial MC-LR induced damage may have been repaired at this time-point and environmental exposures sometimes have a positive effect on cognitive performance^52^. The present study investigated MC-LR’s neurotoxicity in adult post-puberty mice (i.e. older than 8-week-old) for the first time; existing behavioral and neurotoxicity L-BMAA and MC-LR studies in adult animals are listed in Table 1. The positive control experiments using the muscarinic cholinergic antagonist scopolamine confirmed the suitability of using our Barnes maze method to investigate cognitive deficits.

**Table 1:** Animal studies having investigated long-term (>2 weeks after administration) neurotoxic effects for L-BMAA and MC-LR in adult post-puberty (mouse ≥ 8- and rat ≥ 12-week-old) animals.

Substance	Species	Age or weight	Administration	Effect	Reference
L-BMAA	Cynomolgus monkey (macaque, male)	One-year-old (1.4–3.2 kg)	Daily gavage administration of 100–350 mg/kg for 2.5–17 weeks (six differently exposed groups).	Motor-neuron dysfunction after 2–12 weeks. No memory tests performed. Brain and spinal cord structural degenerative changes.	15
L-BMAA	Mouse (CD1, female)	Two-month-old at start	Gavage over 11 weeks (500 mg/kg daily for 18 days, then 500 mg/kg every other day for 28 days, then 1000 mg/kg every other day for 30 days).	No behavioral abnormalities (no specific tests). No pathological or chemical changes observed.	18
L-BMAA	Rat (Sprague-Dawley, male)	200–250 g	Intracerebroventricular administration (500 µg/day) for 16, 30, 40 or 60 days.	Behavioral effects (splay, jerking) for the first six days but which disappeared after 10 days of administration. No memory tests performed.	21
L-BMAA	Mouse (CD1, male)	Six-month-old at start	Through pellet (28 mg/kg BW) daily for 30 days.	Battery of behavioral tests (locomotor, memory (radial arm water maze), etc.) – all were negative. Neuronal cell death in brain and spinal cord analyzed for – also negative.	17
L-BMAA	Rat (Sprague-Dawley, male)	250 g (Adult)	Daily i.v. tail vein injections of 300 mg/kg on three consecutive days.	Behavioral effects (functional disabilities) after two-three weeks. No memory tests performed. Various brain tissue changes.	16
L-BMAA	Monkey (vervet, sex unclear)	Seven-year-old	Dosed fruit, 21 or 210 mg/kg/day for 140 days.	Brain NFT & β-amyloid deposits at both doses. No behavioral testing performed.	19
L-BMAA	Mouse (C57BL/6, male)	∼One-year-old	S.c. injection, 30 mg/kg BW daily for five days.	No behavioral effects observed up to 72 days after administration using Barnes maze, OF, NLR, NOR tests.	This study
**MC-LR, in adult animals**					
MC-LR	Mouse (C57BL/6, male)	∼One-year-old	S.c. injection, 30 µg/kg BW daily for five days.	No behavioral effects observed up to 72 days after administration using Barnes maze, OF, NLR, NOR tests.	This study
**BMAA and MC-LR in combination, in adult animals**					
L-BMAA + MC-LR	Mouse (C57BL/6, male)	∼One-year-old	S.c. injection, 30 µg MC-LR +30 mg L-BMAA/kg BW daily for five days.	No behavioral effects observed up to 72 days after administration using Barnes maze, OF, NLR, NOR tests.	This study

Whereas high doses of L-BMAA has repeatedly been shown to induce acute (within 24 h) transient neurotoxic effects, studies investigating delayed progressive chronic effects (as in ALS-PDC) in animals have often been negative^7^ with the exception of few studies, e.g. the adult monkey study by Spencer from 1987^15^, which has been criticized for excessive doses^7^. Noticeably, all L-BMAA studies in mice have so far been negative regarding both behavioral and mechanistic (e.g. structural) changes, which could have to do with the chosen interspecies dose scaling method (too low doses administrated in rodents)^7^. In adult monkeys, however, a recent L-BMAA study observed neurofibrillary tangles and β-amyloid deposits in the brains, but motor deficits were not reported^19^. Co-administration with L-serine offered protection^19^. Just one previous study^17^ investigated learning/memory (and other behavioral measures) after L-BMAA administration in adult rodents. Similar to our study, no adverse behavioral effects were observed after 30 days repeated oral pellet (28 mg L-BMAA/kg BW/day) administration in six-month-old (at start) CD1 mice. In addition, no neuronal cell death in brain and spinal cord was observed^17^. In young rats, however, high dose L-BMAA administration on postnatal days 9–10 resulted in behavioral effects on motor coordination/posture and learning/memory at adulthood^22,23^. Exposure on postnatal days two and/or five increased OF activity (males only), increased hind limb splay (females only), as well as brain and spinal cord biochemical changes at adulthood^53^. Also high dose L-BMAA administration in juvenile vervet monkeys for 140 days gave neurofibrillary tangles and β-amyloid deposits in the brains^19^. Thus, most rodent studies (except one having administrated repeated high dose intravenous injection in rats over three days^16^) have failed in terms of inducing progressive chronic neurodegeneration from L-BMAA administration, and flying foxes (despite high L-BMAA levels) also show no motor dysfunctions. It may then be expedient to use monkeys (or possibly rats) to mimic ALS-PDC pathology^7^.

No other studies appears to have investigated MC-LR’s neurotoxicity in adult rodents. For young developing animals, however, several studies have observed behavioral and structural/biochemical brain effects (e.g. neurodegeneration)^25–28^ depending on the dose administrated and route of administration. Such effects are likely due to brain MC-LR uptake per se due to hyper-phosphorylation of proteins (e.g. tau hyperphosphorylation which is also seen in Alzheimer’s disease) or oxidative stress (MC-LR appears to be a non-specific metal chelator). Developmental neurotoxic effects may also arise through thyroid hormone system toxicity from MC-LR^54^. There is presently a lack of knowledge regarding MC-LR’s distribution into the brain and its persistence (organ half-life), wherefore a radiolabel (e.g. ^14^C) study would be welcome.

Of the seven quantified parameters in the Barnes maze, scopolamine exerted its greatest effect on escape latency (Fig. 7c) which clearly shows how scopolamine reduced the learning/memory ability, but also the primary latency (particularly days two-three), error numbers (total and primary), and distance travelled were significantly affected parameters. Scopolamine treated animals appeared not to remember how to enter the escape box when they encountered it. This, resulted in a longer distance travelled, more errors and longer latency times. Notably, effects would most likely have been even greater if video recording longer than the maximum 4 min would have been allowed. Interestingly, this is likely the first studies having assessed startdirection error (0° (if the first hole peeped into was the correct hole directly after leaving the transparent cylinder) −180°; 90° on average by random) as a parameter in the Barnes maze, Figs 5a and 7a. As the mice had 30 s to explore the room and look for visual ques located outside the maze when placed in the transparent cylinder, the startdirection error was expected to rapidly decrease with repetition of sessions. However, for both experiments, Figs 5 and 7, there was only a slight (non-significant) improvement in startdirection error over the first three days (six sessions) for unexposed C57BL/6 mice. For the future, it will be interesting to investigate this parameter also for other mouse strains.

Interestingly, adaptive survival tolerability for MC-LR was observed, Fig. 2. Previously, using Swiss Webster mice, significantly increased survival time (and survival) against acute MC-LR lethality was achieved by pre-administration of a sub-lethal MC-LR dose three days prior to administration of a normally lethal high dose^40^. As mechanism, MC-LR may mediate hyperphosphorylation-mediated inactivation of responsible transporters (e.g. OATP)^46^. In addition to self-protection by MC-LR, protection against MC-LR’s lethal effects in rodents was also reported for the flavonoids silybin (silymarin)^36,45,55,56^, quercetin and morin^56^, trypan red which provided protection for three months^42,46^, trypan blue^42,46^, carbon tetrachloride (CCl_4_)^37,42^, pharmacological doses of hydrocortisone^37,42^, vitamin E^45,57^, glutathione^45^, kappa-Selenocarrageenan^58^, phenobarbital^45^, although cyclosporine A^36,42–46^ and rifampin^36,45–47^ offer the greatest protection. Cyclosporin A and rifampin are broad OATP inhibitors^48^, but they also affect expression of numerous metabolic enzymes (e.g. cytochrome P450 enzymes), block hepatocellular uptake of bile acids^45^ (bile acids undergo enterohepatic recirculation), and are immunosuppressors. Overall, this suggests that several different mechanisms can act protectively on the liver, e.g. decreased cellular uptake of MC-LR due to transporter inhibition, upregulation of detoxifying enzymes and/or increased antioxidant defence. Still, repeated (five-day) sub-lethal administration at 30 μg MC-LR/kg BW in the main study resulted in immediate BW loss (liver, and possibly kidney, effects were also observable on day 17 in the dose-range finding study), demonstrating that the mice do not develop a total protection against MC-LR. Some animals (e.g. fish and birds) survive living in cyanobacterial blooms, which raises the question how these animals are adapted to this environment. Antibody recognition ‘immunization’ of MC-LR does most likely not occur due to MC-LR’s small peptide size.

## Conclusions

High dose MC-LR administration reduced BWs temporarily in adult mice, and the dose-range finding study determined a tolerable dose of ~31 μg MC-LR/kg BW for daily s.c. injections for five consecutive days. Tolerability of the first one-two doses was critical. No adverse effects on spatial learning and memory performance were observed in the Barnes maze for either of the cyanobacterial toxins alone or when given as mixture at studied doses. Also, no adverse effects on exploration/anxiety in the OF test or on recollection-like object memory in the NLR & NOR tests were observed. Adult mice on a normal diet appear not to be susceptible to neurodegenerative effects from L-BMAA and MC-LR.

## Materials and Methods

### Ethics statement

Experiments involving live animals were approved by the Norwegian Animal Research Authority (NARA); the ethical animal study permits were FOTS ID 7970 and 7162 (for scopolamine testing). All experiments were performed in accordance with the relevant guidelines and regulations.

### Chemicals

Microcystin-LR (isolated from *Microcystis aeruginosa*, ALX-350–012, ≥95%) was from Enzo Life Sciences, Lausen, Switzerland. L-BMAA hydrochloride (B107, ≥97%) and scopolamine was from Sigma-Aldrich, St. Louis, MO. Due to the high purities, no justification for purity was performed during dose calculations. MC-LR was dissolved in ethanol (1 mg/ml) and diluted in sterile endotoxin-free saline (0.9% NaCl) from B. Braun Melsungen AG (Melsungen, Germany) to 20 µg/ml. L-BMAA was dissolved in saline to 10 mg/ml. Aliquotes of MC-LR and L-BMAA were stored at −80 °C. Scopolamine was dissolved in saline.

### Animals

Male adult C57BL/6JOlaHsd mice (ex-breeders) aged approximately 11 months at toxin administration were ordered from Harlan, the Netherlands. The mice were kept single in cages with free access to RM1 pellets from Special Diet Services, Essex, U.K., and drinking water during controlled conditions (21-23°C, 45-65% RH, 12/12 h light/dark cycle, PET disposable cages, igloos and water bottles (Innovive, CA), 50 ACH positive pressure, aspen bedding (Datesand Ltd., Manchester, U.K.)) at the National Institute of Public Health’s animal facility. Animals in different groups were placed at random on shelves at the animal facility to avoid systematic effects.

### Study design and toxin administration

Two separate toxin studies were performed, see Fig. 1. First, a dose-range study for MC-LR with daily administration once per day over five consecutive days to determine a tolerable dose. On day five, L-BMAA, which is not acutely toxic, was co-administrated at 100 mg/kg BW to check eventual toxicities due to interactions between the two toxins. Based on the determined tolerable dose of MC-LR, a main study investigating long-term behavioral effects was performed. Three days prior to start of administration, solutions to be injected s.c. (1 ml into backs) over five days were prepared based on individual mouse body weight recordings. L-BMAA and MC-LR were diluted (and mixed for one group) in sterile saline, then stored in a refrigerator ( + 6 °C) until and during the five consecutive daily injections. Control mice received 0.9% saline (sterile).

#### Dose-range finding study

Twenty-four mice were randomly divided into eight groups, each consisting of three mice. The mice were administrated 0, 1, 10, 25, 31, 37, 43 or 50 µg MC-LR/kg BW, respectively, once daily for four consecutive days. On the fifth day, in addition to MC-LR, all groups except the control (which received saline only) were co-administered with 100 mg L-BMAA/kg BW (dose was based on studies by others). The mice were observed on a daily basis up to day 17 when sacrificed with collection of organs and blood.

### Sacrifice and organ collection

Mice were injected with 0.2 ml ZRF anesthetic cocktail (routinely prepared at the animal facility containing 3.3 mg zolazepam, 3.3 mg tiletamine, 0.5 mg xylazine, and 2.6 µg fentanyl per ml 0.9% NaCl; stored frozen) before sacrifice. After heart puncture, blood was collected and serum was prepared (blood was allowed to coagulate at room temperature for ~1.5 h, then centrifuged at 1.900 × *g*, +15 °C, 10 min, with collection of the supernatant) and frozen at −80 °C.

### Serum analyses

Serum analyses of the liver status related enzymes alanine aminotransferase (ALT), aspartate aminotransferase (AST) and glutamate dehydrogenase (GD) were performed at Sentrallaboratoriet (Norges veterinærhøgskole), Norwegian University of Life Sciences, Oslo, Norway.

#### Main study

Forty-eight mice were randomly divided into four groups, each consisting of twelve mice. To facilitate handling and testing, the mice were divided into two subsets, each containing half of the mice in each group, with experiments performed one week apart. BWs were checked to be evenly distributed among the groups. For five consecutive days, mice were daily administrated either 30 mg L-BMAA/kg BW, 30 µg MC-LR/kg BW, or a mixture of 30 mg L-BMAA +30 µg MC-LR/kg BW, or only saline.

### Behavioral testing

The mice were tested individually with the video recording and tracking software Ethovision XT from Noldus Inc., Wageningen, the Netherlands.

### Barnes maze

After a latency period of four weeks, spatial learning and memory performance was investigated during three consecutive days using a round light-grey Barnes maze (100 cm in diameter; 20 holes (5 cm in diameter) located around the perimeter 2.5 cm from the edge) from Noldus. A re-test was performed after 10 weeks on one day. Two sessions were performed per day, 4 h in between them. Visual cues were placed on surrounding walls to allow orientation. Each mouse was randomly assigned to a specific ‘escape hole’ having a magnetically attached box beneath it. The location of the escape hole remained constant for the duration of the study in relation to the visual cues. The maze was illuminated with 440–590 (border) to 830–900 (center) lux. Mice (individual testing) were held for 30 s inside a transparent cylinder positioned in the center of the maze. After removal of the cylinder, the mice were maximally allowed 4 min in the maze. Mice that did not enter the escape box were gently guided into the escape tunnel. To increase the motivation a fan blew air onto the maze on day three (week four) and during the re-test (week 10). The maze, the cylinder and the escape box were carefully cleaned using water and allowed to dry between each trial. The maze was turned 90° clock-wise between each session. The testing was done in the animal’s light cycle, between 09.00 a.m. and 04.00 p.m. The following parameters were analyzed either manually or using the video tracking software Ethovision XT: startdirection error (0 to 180 degrees - first peek into a whole in relation to the correct hole), primary latency (s - time to the first peek into the correct hole), escape latency (s – time to enter the correct hole with all four feet), number of primary errors (number of peeks into wrong holes before peeking into the correct hole), number of total errors (number of peeks into wrong holes before entering the correct hole), mean velocity (cm/s), and distance travelled (m).

### OF, NLR and NOR

The OF, NLR and NOR tests were performed eight weeks after administration inside four identical square-shaped plastic boxes (each 50 × 50 cm with light-grey bottoms and white walls) illuminated at 70–130 lux, allowing simultaneous video-recording of the boxes from above. The testing procedures followed those by Seigers *et al*.^50^. On the first day, exploration, anxiety and general locomotor activity was investigated using the OF test which lasted 10 min. Time spent in the center 25 × 25 cm squared zone (a quarter of the total area) was analyzed. Prior to OF trial recordings, mice were allowed to observe the box for 30 s through transparent cylinders (inner diameter 8 cm) which had been placed inside the boxes and which were simultaneously removed when starting the trial. After each trial, the boxes (and objects) were carefully cleaned using 70% ethanol.

On the following day (after 24 h), two gold-colored wood cubes (3.2 × 3.2 × 3.2 cm) were placed in opposite corners (12.5 cm from the walls) and a 5 min acquisition session followed (no analysis). Then, willingness to explore as well as hippocampal and peri-postrhinal cortex dependent recollection-like object memory was investigated using NLR and NOR tests that lasted 5 min each. After 2 h, one of the cubes was moved so that the two cubes were located on the same side of the box, but in opposite corners. The area (diameter 12.5 cm) around the stationary cube was designated zone 1 in Ethovision XT, and the corresponding area around the moved object zone 2. The NLR test was now performed with analyses of the number of entries into the two zones as well as how much time the mouse spent facing the respective object inside the zones. Finally, after additional 2 h, the cube which had been moved (located in zone 2) was replaced with an up-right wood cylinder (3.2 cm long, 2.5 cm diameter) of identical golden color, and the NOR test was performed with comparison of interest for the familiar (zone 1) and novel (zone 2) objects. Prior to NLR and NOR trial recordings, mice were placed in covered cylinders for 30 s inside the boxes and that were removed when starting the trial.

#### Scopolamin study

Scopolamine was used as a positive control to investigate its effect on the seven quantified Barnes maze parameters. Similar to a previous study^49^ in which two Barnes maze parameters were quantified, scopolamine was injected as 1 mg/kg BW i.p. and compared to mice injected with vehicle (saline) alone. Fourteen male C57BL/6NTac (transgenic Ogg1^-^/^-^ homozygotes) mice aged four-nine months (25.0–30.9 g) were randomly divided into two group. Scopolamine was diluted to 0.1 mg/ml saline. Fifteen min before each Barnes maze trial, each mouse received scopolamine (1 mg/kg BW; e.g. 280 µl/28 g BW) or an equal amount of saline. Testing procedures followed those described above. To increase the motivation a fan blew air onto the maze on day three (both sessions).

#### Statistical methods

Statistical analyses were performed using JMP pro 13 from SAS institute, Cary, NC. Serum, organ and BW data were analyzed using linear regression and one-way ANOVA. Barnes maze generated data were analyzed for effects between the groups (comparison of curves) using repeated measures (repeated factor: training session) ANOVA, and, using two-way ANOVA and Dunnett’s *t*-test (comparison against the control group) at individual sessions. OF, NLR and NOR data were analyzed using one-way ANOVA. Graphs are presented using Prism 5 from Graph Pad Software, San Diego, CA.

## Electronic supplementary material

Supplementary Information
