# Supplementary material for: Repeated five-day administration of L-BMAA, microcystin-LR, or as mixture, in adult C57BL/6 mice - lack of adverse cognitive effects
Source: Sci Rep. 2018 Feb 2;8:2308. doi: 10.1038/s41598-018-20327-y (PMC5797144; doi:10.1038/s41598-018-20327-y)
Supplement: Supplementary file 1 — Supplementary Information [file 41598_2018_20327_MOESM1_ESM.pdf]

Repeated five-day administration of L-BMAA, microcystin-LR, or as mixture, in adult C57BL/6 mice - lack of adverse cognitive effects

Oddvar Myhre, Dag Marcus Eide, Synne Kleiven, Hans Christian Utkilen, Tim Hofer

Supplementary Table S1. BW (g) recordings in the dose-range finding study. BWs five days prior to start of administration were used to calculate the concentration of MC-LR to be subcutaneously injected on days 1-5 (study start: day 1). Mice were observed until day 17 when they were sacrificed.

			Administration day										
Animal ID	MC-LR (µg/kg BW/day)	-5	1	2	3	4	5	8	10	12	15	17	
1A	10	26.2	25.3	25.3	25.4	26.0	26.1		26.4			25.7	
1B	10	26.0	24.8	24.6	24.3	26	26.0		25.5			25.7	
1C	10	27.1	25.5	25.5	25.4	25.8	26.2		25.8			24.5	
2A	1	26.0	26.1	26.0	25.8	26.1	27.1		27.0			25.9	
2B	1	26.3	27.9	28.0	28.1	28.6	29.0		28.3			28.6	
2C	1	28.0	28.0	28.5	28.4	28.4	28.1		26.5			27.3	
3A	25	27.5	26.2	24.0	23.5	22.6	23.1		-			26.0	recording mistake
3B	25	27.3	27.2	27.1	27.1	26.0	26.6		-			-	recording mistake
3C	25	27.9	27.6	27.2	26.8	25.6	25.8		-			-	recording mistake
4A	50	28.6	28.5	†									
4B	50	27.5	26.4	26.0	†								
4C	50	29.8	27.1	†									
5A	43	27.2	27.5	26.9	26.6	26.4	26.4	26.5	26.4	26.2	26.7	27.4	
5B	43	29.8	29.2	†									
5C	43	28.8	29.5	28.4	28.0	27.3	27.4	27.4	27.4	27.2	27.2	27.2	
6A	37	31.4	31.4	†									
6B	37	28.7	29.1	28.7	28.0	27.4	27.4	27.6	27.5	27.5	29.1	28.8	
6C	37	30.9	32.0	31.1	29.9	29.8	29.7	29.1	28.3	27.9	28.6	29.6	
7A	31	29.2	29.9	29.3	28.9	28.5	28.4	28.2	28.1	27.8	27.9	28.3	
7B	31	28.1	28.7	28.8	28.1	27.7	27.9	28.4	29.0	29.2	29.1	29.9	
7C	31	29.2	29.6	29.4	29.0	28.2	28.3	28.7	28.7	28.7	30.1	30.6	
8A	0	32.6	34.5	33.9	33.3	33.1	32.3	31.6	32.0	32.4	33.5	33.5	
8B	0	29.0	29.6	28.8	29.3	29.2	29.0	28.9	28.7	28.6	29.3	29.3	
8C	0	37.2	38.9	38.1	37.4	37.1	37.2	37.4	37.4	37.2	38.4	39.1	

Supplementary Fig. S1. Normalized BWs (%) recordings in the dose-range finding study.

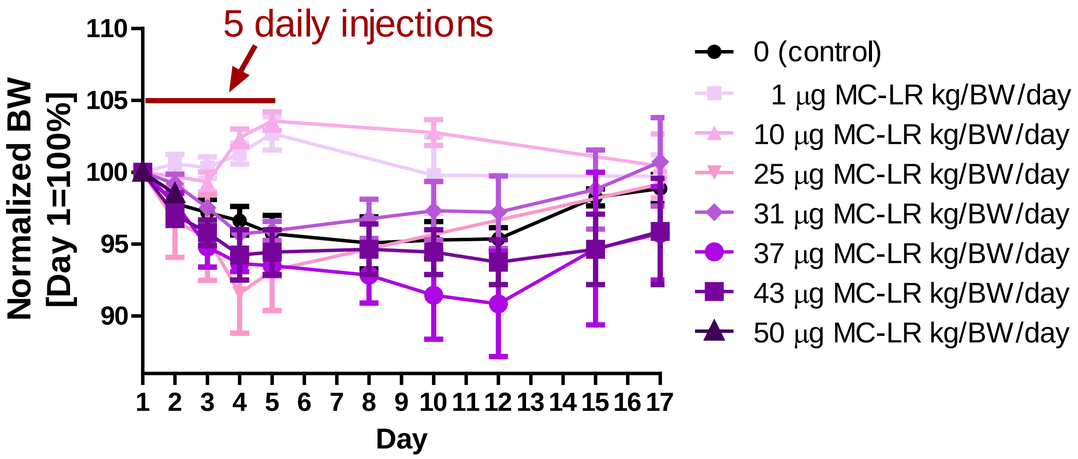

Supplementary Table S2. Organ weights (g) at sacrifice (day 17) in the dose-range finding study.

Animal ID	MC-LR (µg/kg BW/day)	Liver (g)	Kidneys (both) (g)	BW (g)	Liver/BW	Kidneys/BW	
1A	10	1.0322	0.3122	25.7	0.04016	0.01215	
1B	10	1.3135	0.2855	25.7	0.05111	0.01111	
1C	10	1.0141	0.2654	24.5	0.04139	0.01083	
2A	1	1.1758	0.2862	25.9	0.04540	0.01105	
2B	1	1.3094	0.3109	28.6	0.04578	0.01087	
2C	1	1.2562	0.3036	27.3	0.04601	0.01112	
3A-C	25	-	-	-	-	-	recording mistake
4A-C	50						†††
5A	43	1.5448	0.3455	27.4	0.05638	0.01261	
5B	43						†
5C	43	1.275	0.3219	27.2	0.04688	0.01183	
6A	37						†
6B	37	1.4383	0.3592	28.8	0.04994	0.01247	
6C	37	1.535	0.3841	29.6	0.05186	0.01298	
7A	31	1.3617	0.3486	28.3	0.04812	0.01232	
7B	31	1.4594	0.3456	29.9	0.04881	0.01156	
7C	31	1.6143	0.3549	30.6	0.05275	0.01160	
8A	0	1.4774	0.3672	33.5	0.04410	0.01096	
8B	0	1.4172	0.3434	29.3	0.04837	0.01172	
8C	0	1.9516	0.3861	39.1	0.04991	0.009875	

Supplementary Table S3. Serum levels of liver status enzymes (U/L) at sacrifice (day 17) in the dose-range finding study. ALT=Alanine aminotransferase, AST=aspartate aminotransferase, GD=glutamate dehydrogenase.

Animal ID	MC-LR (µg/kg BW/day)	ALT (U/L)	AST (U/L)	GD (U/L)	
1A	10	30	140	6	
1B	10	34	96	6	
1C	10	39	189	7	
2A	1	39	145	7	
2B	1	39	153	6	
2C	1	42	131	6	
3A	25	40	134	7	
3B	25	40	139	9	
3C	25	42	103	7	
4A-C	50				†††
5A	43	74	109	8	
5B	43				†
5C	43	143	134	40	
6A	37				†
6B	37	37	90	6	
6C	37	109	108	22	
7A	31	55	95	13	
7B	31	33	86	5	
7C	31	40	84	4	
8A	0	39	90	6	
8B	0	42	168	6	
8C	0	41	136	6	

Supplementary Table S4. BW (g) recordings in the main study. BWs seven (subset 1 of 2) or five (subset 2 of 2) days prior to start of administration was used to calculate the concentration of MC-LR to be subcutaneously injected on days 1-5 (study start: day 1).

				Administration day																	
	Animal ID	-7	-5	1	2	3	4	5	8	10	12	15	17	19	22	24	38	45	52	66	72
30 µg MC-LR/kg BW/day (N=12)	9A	25.7		26.4	27.1	25.6	25.4	25.6	24.9	24.9	25.4	26.0	25.6	26.0	26.5	27.2	27.7	25.5	26.6	27.8	28.4
	9B	27.7		28.8	29.3	28.7	26.9	27.1	27.9	27.4	27.9	28.3	28.5	29.0	29.8	29.0	29.2	30.0	30.1	29.4	29.4
	9C	30.4		29.3	29.5	28.5	27.8	28.3	28.0	28.0	28.0	28.2	28.4	29.0	29.5	29.1	29.8	30.4	30.7	31.9	31.9
	9D	31.3		31.9	31.5	31.2	30.4	29.2	29.1	29.1	28.0	27.6	27.8	28.0	28.6	29.2	29.1	29.1	30.6	31.8	29.9
	9E	23.9		28.2	29.6	28.8	28.4	27.4	27.9	27.8	28.1	27.7	28.2	29.0	29.0	28.7	29.6	29.8	31.6	32.1	31.3
	9F	33.2		32.1	31.8	31.5	31.4	30.9	29.5	30.0	29.6	30.6	30.3	30.5	30.6	31.5	32.4	33.0	32.9	34.4	33.7
	9G		28.9	29.7	28.7	28.6	27.3	28.9	28.6	28.5	29.0	29.2	29.6			29.6	30.0	30.2	30.9		30.3
	9H		28.8	28.6	28.2	28.5	28.8	28.7	27.6	27.1	27.5	27.7	27.6			29.0	28.8	28.7	28.9		29.6
	9I		27.4	27.5	26.4	27.0	26.5	26.1	26.4	26.7	26.4	26.0	26.3			28.2	27.2	28.0	27.9		27.4
	9J		26.7	26.5	27.1	26.8	25.7	24.9	25.6	26.5	26.4	26.2	27.4			27.0	27.0	27.2	27.0		27.0
	9K		29.8	29.9	28.4	29.0	27.3	27.5	27.7	26.8	27.9	28.7	28.9			29.1	28.8	28.9	28.7		29.7
	9L		29.0	28.4	28.2	28.0	27.0	26.8	28.6	27.6	27.6	27.5	27.3			28.0	27.8	27.9	28.0		27.8
30 mg L-BMAA/kg BW/day (N=12)	10A	33.8		31.1	33.1	33.5	34.0	33.5	33.5	33.3	33.1	33.3	33.4	33.2	33.1	32.6	34.0	33.5	33.9	33.9	33.3
	10B	26.9		25.5	26.8	26.8	26.9	27.0	26.1	26.8	26.4	26.0	25.6	26.0	26.7	27.1	26.6	27.6	27.7	27.1	26.7
	10C	34.7		35.8	35.8	35.6	35.1	34.6	34.4	35.0	36.1	36.5	36.2	36.4	36.4	37.2	37.4	39.7	38.8	40.9	41.3
	10D	24.6		27.3	27.6	26.5	27.2	27.6	26.7	27.0	26.8	27.7	28.0	27.7	27.4	28.1	29.0	28.5	27.9	27.7	27.8
	10E	26.8		28.7	28.3	27.7	27.9	27.6	28.2	28.2	27.2	27.9	27.9	28.0	28.4	28.4	27.4	28.8	29.6	26.4	29.4
	10F	29.1		29.2	29.6	29.6	29.0	29.7	29.7	29.8	29.9	29.7	30.1	30.1	30.1	30.7	31.4	32.3	32.0	32.0	31.9
	10G		27.8	27.8	27.7	27.7	27.3	27.7	27.1	27.0	28.0	28.1	27.7			28.0	28.0	28.1	28.0		28.6
	10H		27.2	27.1	27.2	27.0	27.1	26.9	27.1	26.9	27.0	27.4	27.2			27.2	27.0	27.0	27.1		27.5
	10I		29.5	29.8	30.4	29.9	29.3	30.4	29.5	30.5	30.7	30.7	31.0			32.0	30.6	30.0	30.0		30.8
	10J		28.5	28.0	27.3	27.8	27.2	27.1	28.1	27.9	28.0	28.3	28.6			28.6	28.3	29.0	28.7		29.3
	10K		32.2	34.0	33.8	33.9	33.6	33.4	33.4	33.9	33.5	33.5	34.3			35.9	34.9	34.1	34.7		36.0
	10L		30.5	31.0	30.6	30.9	31.9	31.1	30.6	31.5	32.0	32.5	33.0			34.5	32.4	33.6	33.0		33.7
30 µg MC-LR +30 mg L-BMAA/kg BW/day (N=12)	11A	29.0		27.8	27.2	26.7	26.5	25.9	26.2	26.2	26.7	26.5	27.0	27.8	28.1	27.3	27.9	28.2	28.4	27.7	27.5
	11B	31.7		30.1	30.0	29.7	29.3	28.9	27.5	27.8	26.8	26.9	27.0	27.5	27.8	27.8	28.5	30.2	29.9	28.3	28.6
	11C	34.1		35.3	36.3	35.2	34.8	34.3	34.2	34.1	34.8	35.6	35.7	35.2	35.0	35.7	36.0	38.0	38.1	39.5	38.8
	11D	29.2		28.3	28.1	27.9	28.5	26.8	28.0	27.9	26.9	27.8	28.6	28.3	28.3	28.7	30.2	29.8	29.7	29.9	29.1

	11E	27.3		27.5	26.5	26.6	26.6	26.5	25.4	25.9	26.3	25.1	25.8	26.0	26.6	26.5	27.6	26.7	27.1	27.1	27.2
	11F	29.2		29.2	28.6	27.6	28.1	28.3	26.8	27.1	27.3	28.2	27.6	28.0	28.6	29.2	29.0	29.7	30.7	30.2	29.6
	11G		28.2	28.7	27.9	27.5	26.5	25.9	28.1	27.9	28.0	28.2	28.3			29.0	29.0	29.2	29.7		28.6
	11H		27.8	28.7	27.4	27.9	26.0	27.7	26.5	27.0	27.2	27.1	27.7			27.9	27.6	27.9	27.9		28.0
	11I		30.6	30.8	30.0	30.7	28.0	27.6	28.8	29.1	30.1	30.4	30.5			30.6	30.9	30.7	31.1		30.7
	11J		29.5	30.2	29.3	30.1	28.2	28.0	28.7	28.0	28.0	28.1	29.2			28.7	28.8	28.6	28.7		28.2
	11K		29.5	29.2	29.1	29.0	28.4	27.7	28.8	29.2	29.2	29.1	28.9			29.1	30.1	30.0	29.2		30.7
	11L		26.8	27.4	27.5	27.3	25.9	25.0	26.9	26.6	26.7	26.7	26.3			27.6	26.8	28.3	28.0		27.1
Control, saline only (N=12)	12A	32.3		33.2	33.2	33.5	33.1	32.9	32.8	32.9	33.6	34.3	34.2	34.2	34.6	35.4	34.2	34.2	34.4	31.8	31.7
	12B	32.9		29.2	30.1	30.5	29.7	30.1	29.4	30.0	30.4	30.2	29.4	29.4	29.9	29.6	29.7	31.3	28.8	28.3	28.1
	12C	31.6		29.8	29.4	29.3	29.4	29.5	29.6	29.5	29.6	29.6	29.9	29.4	29.4	28.8	30.0	30.5	29.9	30.0	30.0
	12D	28.5		28.5	28.1	28.6	27.6	28.1	28.7	28.2	29.0	29.1	28.4	28.4	28.5	28.7	27.8	28.1	28.1	28.5	28.6
	12E	25.8		24.9	25.3	25.8	25.7	25.6	25.7	25.9	26.3	26.6	26.8	26.8	26.9	26.9	27.1	27.6	27.4	27.2	27.2
	12F	29.8		29.1	28.7	29.5	29.3	29.5	29.3	29.2	29.7	29.2	29.3	29.3	29.1	28.7	28.2	28.1	29.0	29.5	29.4
	12G		29.3	29.4	29.4	29.3	28.9	29.2	30.2	30.4	29.9	29.8	30.1			30.0	29.7	30.1	30.5		30.4
	12H		33.6	34.6	34.9	34.8	35.2	35.1	35.8	36.4	36.4	36.2	37.4			37.2	37.4	37.4	38.0		39.4
	12I		28.2	28.9	28.4	28.4	27.8	28.1	28.3	27.6	27.7	27.8	27.8			28.5	28.1	28.4	28.5		28.4
	12J		28.9	28.8	28.0	28.6	27.5	27.9	27.4	27.2	28.0	28.1	28.0			28.1	27.7	28.0	28.4		27.0
	12K		27.8	28.2	28.1	28.3	28.1	27.9	27.8	28.8	28.7	28.2	28.5			28.5	28.1	29.0	29.4		29.6
	12L		33.3	32.6	32.9	32.9	33.0	33.4	33.3	33.2	33.4	33.6	33.5			34.0	33.0	33.3	33.0		32.9

Supplementary Table S5. Organ weights (g) in the main study at sacrifice (day 72).

	Animal ID	Liver (g)	Kidneys (both) (g)	BW (g)	Liver/BW	Kidneys/BW
30 µg MC-LR/kg BW/day (N=12)	9A	1.4271	0.3175	28.4	0.05025	0.01118
	9B	1.3654	0.3090	29.4	0.04644	0.01051
	9C	1.4208	0.3152	31.9	0.04454	0.00988
	9D	1.2842	0.3265	29.9	0.04295	0.01092
	9E	1.4088	0.3227	31.3	0.04501	0.01031
	9F	1.4495	0.3338	33.7	0.04301	0.00991
	9G	1.3685	0.3129	30.3	0.04517	0.01033
	9H	1.4765	0.3200	29.6	0.04988	0.01081
	9I	1.3652	0.3003	27.4	0.04982	0.01096
	9J	1.4057	0.3127	27.0	0.05206	0.01158
	9K	1.3567	0.3134	29.7	0.04568	0.01055
	9L	1.1987	0.2880	27.8	0.04312	0.01036
30 mg L-BMAA/kg BW/day (N=12)	10A	1.4722	0.3428	33.3	0.04421	0.01029
	10B	1.3126	0.2770	26.7	0.04916	0.01037
	10C	1.9306	0.3519	41.3	0.04675	0.00852
	10D	1.3480	0.3217	27.8	0.04849	0.01157
	10E	1.5560	0.3118	29.4	0.05293	0.01061
	10F	1.4050	0.3265	31.9	0.04404	0.01024
	10G	1.3391	0.3036	28.6	0.04682	0.01062
	10H	1.4700	0.2843	27.5	0.05345	0.01034
	10I	1.4384	0.3038	30.8	0.04670	0.00986
	10J	1.4531	0.3110	29.3	0.04959	0.01061
	10K	1.7282	0.3370	36.0	0.04801	0.00936
	10L	1.7770	0.3558	33.7	0.05273	0.01056
30 µg MC-LR +30 mg L-BMAA/kg BW/day (N=12)	11A	1.2851	0.3234	27.5	0.04673	0.01176
	11B	1.3774	0.3168	28.6	0.04816	0.01108
	11C	1.7502	0.3868	38.8	0.04511	0.00997
	11D	1.4060	0.3027	29.1	0.04832	0.01040
	11E	1.3356	0.2717	27.2	0.04910	0.00999
	11F	1.3713	0.3151	29.6	0.04633	0.01065
	11G	1.4513	0.3245	28.6	0.05074	0.01135
	11H	1.4490	0.2687	28.0	0.05175	0.00960
	11I	1.2323	0.3282	30.7	0.04014	0.01069
	11J	1.4898	0.3062	28.2	0.05283	0.01086
	11K	1.5154	0.3077	29.6	0.05120	0.01040
	11L	1.3518	0.2681	27.1	0.04988	0.00989
Control, saline only (N=12)	12A	1.5758	0.3516	31.7	0.04971	0.01109
	12B	1.3275	0.2961	28.1	0.04724	0.01054
	12C	1.4505	0.3283	30.0	0.04835	0.01094
	12D	1.0664	0.2898	25.6	0.04166	0.01132
	12E	1.3114	0.3124	27.2	0.04821	0.01149
	12F	1.4681	0.2986	29.4	0.04994	0.01016
	12G	1.5068	0.3535	30.4	0.04957	0.01163
	12H	1.8904	0.3625	39.4	0.04798	0.00920
	12I	1.4522	0.3077	28.4	0.05113	0.01083

12J	1.2480	0.2941	27.0	0.04622	0.01089
12K	1.4300	0.3240	30.7	0.04658	0.01055
12L	1.5569	0.3349	32.9	0.04732	0.01018

Supplementary Table S6. Barnes maze startdirection error (0 to 180 degrees) data in the main study.

		Session number (W=week; D=day; '/''=session 1&2)							
	Animal ID	W4D1'	W4D1''	W4D2'	W4D2''	W4D3'	W4D3''	W10D1'	W10D1''
30 µg MC-LR/kg BW/day (N=12)	9A	72	162	72	72	126	108	72	36
	9B	72	180	54	54	72	18	90	54
	9C	0	36	126	36	90	126	108	180
	9D	144	144	54	144	144	126	108	18
	9E	18	18	36	18	18	0	0	126
	9F	54	108	54	72	126	18	36	90
	9G	90	108	144	108	72	162	162	54
	9H	72	90	108	162	18	18	126	90
	9I	72	0	18	162	72	18	0	18
	9J	72	162	18	36	18	126	54	90
	9K	144	18	18	180	72	162	180	126
	9L	90	108	126	126	126	108	162	144
30 mg L-BMAA/kg BW/day (N=12)	10A	126	108	18	36	162	18	126	126
	10B	144	180	108	162	144	144	108	126
	10C	36	72	144	54	18	36	18	0
	10D	0	18	36	36	18	54	72	144
	10E	54	144	72	126	54	144	162	36
	10F	72	162	90	108	108	36	36	54
	10G	72	180	72	144	108	18	126	108
	10H	54	36	54	36	36	36	36	36
	10I	126	90	90	18	144	0	180	162
	10J	180	0	180	90	144	90	162	144
	10K	36	36	18	90	18	0	18	144
	10L	90	54	54	108	72	144	126	180
30 µg MC-LR +30 mg L-BMAA/kg BW/day (N=12)	11A	36	144	36	36	0	18	36	0
	11B	126	162	36	162	108	144	162	126
	11C	36	0	0	54	0	18	36	0
	11D	36	54	18	72	0	36	0	36
	11E	126	144	162	0	36	90	144	36
	11F	162	144	36	36	0	0	144	72
	11G	180	162	144	18	180	180	54	144
	11H	126	108	18	36	36	18	72	54
	11I	144	90	36	162	162	90	54	162
	11J	72	18	72	36	90	54	18	72
	11K	18	36	108	18	36	18	0	0
	11L	54	36	0	90	0	72	90	18
Control, saline only (N=12)	12A	144	36	18	18	0	36	36	126
	12B	108	72	162	90	180	90	162	126
	12C	72	36	36	126	108	126	108	0
	12D	108	90	180	90	144	18	162	162
	12E	72	72	126	36	54	36	72	0

12F	144	144	162	18	0	18	144	144
12G	0	0	0	0	0	18	54	0
12H	108	54	72	72	54	108	90	36
12I	108	18	0	144	72	54	108	54
12J	54	72	72	90	90	36	90	90
12K	144	180	180	162	180	162	180	180
12L	36	18	18	36	0	18	18	144

Supplementary Table S7. Barnes maze primary latency (s) data in the main study.

		Session number (W=week; D=day; '/''=session 1&2)							
	Animal ID	W4D1'	W4D1''	W4D2'	W4D2''	W4D3'	W4D3''	W10D1'	W10D1''
30 µg MC-LR/kg BW/day (N=12)	9A	240	240	240	12.32	240	11.52	144.88	112.08
	9B	191.84	161.12	20.64	36.48	80.64	4.96	37.12	6.88
	9C	11.44	97.12	127.60	12.84	10.88	9.12	17.60	22.24
	9D	61.12	240	221.92	210.32	238.96	26.80	18.08	4.08
	9E	219.52	7.68	240	5.44	174.08	2.88	2.88	13.44
	9F	240	198.40	63.60	24.96	12.32	152.16	161.68	100.32
	9G	137.12	109.36	20.88	18.88	21.44	19.04	35.76	240
	9H	240	152.08	95.36	24.32	11.92	6.40	17.60	68.64
	9I	225.36	5.12	5.36	67.68	15.84	24.24	9.76	3.76
	9J	240	17.20	126.64	3.60	10.48	14.80	43.92	9.36
	9K	240	9.28	17.60	170.48	74.56	80.40	34.40	29.04
	9L	186.00	182.08	56.24	14.48	15.20	9.44	22.80	10.64
30 mg L-BMAA/kg BW/day (N=12)	10A	40.40	21.76	7.6	11.44	36.72	31.04	27.04	19.28
	10B	21.92	47.44	10.72	39.36	99.04	14.40	31.12	15.12
	10C	52.80	45.44	52.48	16.00	3.28	7.76	6.96	6.80
	10D	4.32	137.20	62.56	69.70	8.80	43.60	31.12	12.24
	10E	240	240	175.84	71.04	32.40	27.12	210.80	175.28
	10F	175.68	29.44	14.48	10.88	9.92	24.72	24.16	105.12
	10G	32.80	66.64	145.12	22.80	50.08	14.72	213.12	78.16
	10H	20.16	12.80	60.40	17.68	18.96	133.04	14.08	17.20
	10I	240	186.96	188.80	6.32	99.68	4.24	22.56	224.72
	10J	30.16	2.56	117.68	31.76	31.28	28.24	37.92	26.16
	10K	18.00	16.32	80.48	23.76	5.28	3.28	3.92	98.16
	10L	62.08	240	75.52	75.44	23.04	29.92	14.08	24.08
30 µg MC-LR +30 mg L-BMAA/kg BW/day (N=12)	11A	235.84	21.68	11.68	6.08	4.16	39.68	39.52	8.00
	11B	240	217.04	71.74	91.76	26.32	9.20	73.28	44.56
	11C	34.72	6.16	5.68	17.76	7.04	23.36	12.56	3.20
	11D	240	240	24.24	26.08	6.24	16.16	4.24	12.08
	11E	240	220.16	35.53	4.24	16.80	19.20	140.96	10.32
	11F	172.72	72.32	11.84	10.48	3.92	2.48	17.60	37.76
	11G	240	22.56	240	240	127.04	64.00	81.84	35.20
	11H	26.24	73.76	79.52	80.96	7.76	6.16	39.76	32.48
	11I	240	240	95.36	25.84	28.56	28.56	13.28	41.04
	11J	151.76	7.12	54.08	33.52	20.32	5.76	26.08	50.64
	11K	18.32	131.52	240	18.40	78.56	53.76	2.72	3.36
	11L	19.20	154.16	3.36	14.96	9.28	57.28	13.08	26.56
	12A	197.12	43.12	9.76	8.56	8.88	24.32	18.72	48.72

	12B	240	12.64	74.48	33.84	21.68	12.16	186.88	26.48
	12C	40.40	216.24	41.28	18.32	20.72	52.24	183.04	2.72
	12D	154.00	29.84	18.56	71.76	196.56	6.48	50.96	36.88
	12E	240	79.92	97.52	14.56	240	38.40	46.24	4.08
	12F	240	240	144.32	29.36	10.48	18.40	240	240
Control, saline only (N=12)	12G	6.64	7.76	5.28	6.56	5.20	4.80	3.04	16.88
	12H	240	189.36	100.32	36.16	12.64	13.20	144.08	8.24
	12I	44.8	20.40	28.48	176.00	240	26.24	240	46.24
	12J	240	191.76	21.84	11.76	11.12	10.80	41.20	12.32
	12K	180.32	40.08	25.92	14.72	40.00	10.96	95.44	12.16
	12L	240	10.56	146.48	12.40	2.80	4.16	8.00	32.56

Supplementary Table S8. Barnes maze escape latency (s) data in the main study.

		Session number (W=week; D=day; '/''=session 1&2)							
	Animal ID	W4D1'	W4D1''	W4D2'	W4D2''	W4D3'	W4D3''	W10D1'	W10D1''
30 µg MC-LR/kg BW/day (N=12)	9A	240	240	240	61.84	240	13.92	153.04	116.08
	9B	240	240	107.60	43.20	87.36	7.52	53.04	12.16
	9C	240	158.40	129.36	15.52	12.80	10.80	69.84	28.56
	9D	240	240	240	240	240	36.64	37.68	7.12
	9E	240	240	240	8.40	179.92	5.92	10.48	18.32
	9F	240	172.24	78.08	27.60	17.60	158.56	165.52	108.56
	9G	240	240	35.52	33.20	28.40	27.28	65.36	240
	9H	240	166.08	109.68	27.36	14.96	9.28	23.28	70.96
	9I	240	240	10.00	69.92	17.44	29.76	17.04	6.88
	9J	240	29.04	139.76	6.40	13.52	17.52	45.92	10.72
	9K	240	52.56	29.04	188.16	76.48	83.36	41.36	32.40
	9L	240	208.00	65.20	21.76	17.76	11.84	25.28	12.32
30 mg L-BMAA/kg BW/day (N=12)	10A	240	178.56	87.12	78.64	40.72	36.40	49.12	25.52
	10B	240	240	150.80	43.84	104.08	19.84	33.20	16.32
	10C	240	51.20	78.24	22.56	8.00	9.52	17.12	11.12
	10D	240	154.16	64.32	75.60	12.32	49.20	106.96	14.32
	10E	240	240	184.08	75.44	37.36	28.72	240	186.00
	10F	240	240	159.28	30.48	15.12	30.80	33.12	106.96
	10G	240	75.84	150.56	25.60	53.28	16.96	227.60	87.68
	10H	240	240	75.36	26.88	22.24	142.96	17.60	20.88
	10I	240	240	198.24	-	103.84	6.72	240	226.40
	10J	240	35.28	139.28	34.96	35.44	31.04	47.04	27.92
	10K	240	49.80	85.04	28.00	9.76	5.28	10.00	124.96
	10L	240	240	88.72	82.72	25.12	32.80	25.60	27.28
30 µg MC-LR +30 mg L-BMAA/kg BW/day (N=12)	11A	240	211.04	16.24	37.12	10.24	42.16	46.48	10.00
	11B	240	225.52	78.88	110.04	33.20	21.60	83.12	47.04
	11C	198.78	42.88	70.88	31.12	11.92	31.12	16.08	9.12
	11D	240	240	25.68	41.12	12.88	19.20	9.60	14.08
	11E	240	240	118.80	10.48	18.16	20.16	148.08	17.52
	11F	240	103.44	14.96	16.24	10.08	6.72	19.28	42.72
	11G	240	240	240	240	141.52	70.88	83.52	39.60
	11H	240	240	95.52	90.24	14.08	9.36	43.04	34.64
	11I	240	240	134.88	30.80	29.92	32.16	23.60	43.04

	11J	240	78.96	194.16	37.52	22.88	11.36	31.76	51.76
	11K	240	141.52	240	31.92	82.56	55.60	10.96	5.44
	11L	240	174.08	87.52	25.52	11.52	64.08	17.08	28.72
Control, saline only (N=12)	12A	240	49.52	12.16	14.80	11.44	25.68	22.80	50.48
	12B	240	200.16	82.32	40.88	25.84	17.76	207.20	34.24
	12C	240	227.92	58.00	26.88	24.40	54.48	188.72	5.60
	12D	240	144.48	26.08	78.80	199.92	7.92	54.00	39.84
	12E	240	240	240	240	240	48.48	121.20	12.64
	12F	240	240	151.52	32.08	12.00	19.76	240	240
	12G	240	97.76	9.44	14.72	10.32	9.36	8.40	18.88
	12H	240	207.60	116.32	42.48	15.44	15.04	152.32	9.84
	12I	240	240	206.64	195.04	240	30.72	240	51.44
	12J	240	198.08	30.32	20.64	16.00	16.00	43.76	15.76
	12K	240	87.52	28.88	16.32	41.52	12.40	104.88	18.24
	12L	240	101.68	162.56	-	10.96	7.12	29.76	35.20

Supplementary Table S9. Barnes maze number of primary errors data in the main study.

		Session number (W=week; D=day; '/''=session 1&2)							
	Animal ID	W4D1'	W4D1''	W4D2'	W4D2''	W4D3'	W4D3''	W10D1'	W10D1''
30 µg MC-LR/kg BW/day (N=12)	9A	11	6	8	1	9	3	16	5
	9B	28	23	6	9	12	1	10	3
	9C	0	3	14	2	3	6	4	5
	9D	5	17	19	9	15	7	6	1
	9E	16	1	16	1	18	0	0	2
	9F	19	14	4	4	2	12	10	10
	9G	16	17	4	2	4	5	6	17
	9H	7	4	4	6	1	1	5	14
	9I	13	0	1	16	3	7	3	0
	9J	20	1	14	1	1	3	10	3
	9K	26	1	1	21	9	6	7	6
	9L	23	17	16	7	3	4	9	3
30 mg L-BMAA/kg BW/day (N=12)	10A	6	6	1	2	9	15	9	7
	10B	2	7	3	9	14	6	9	3
	10C	8	6	8	3	1	2	1	0
	10D	0	11	11	11	1	16	14	4
	10E	1	12	8	10	2	5	20	10
	10F	36	7	6	6	3	6	8	16
	10G	6	9	18	4	15	4	16	15
	10H	3	1	8	3	2	10	3	4
	10I	17	9	8	1	9	0	4	20
	10J	5	0	29	9	8	13	10	9
	10K	3	4	6	8	1	0	1	29
	10L	12	19	16	9	4	7	4	10
30 µg MC-LR +30 mg L-BMAA/kg BW/day (N=12)	11A	19	4	2	1	0	8	11	0
	11B	24	19	5	16	6	1	13	8
	11C	5	0	0	3	0	6	2	0
	11D	20	13	4	4	0	4	0	2
	11E	9	19	2	0	2	3	10	2

	11F	30	16	1	2	0	0	4	8
	11G	18	3	32	7	12	11	20	14
	11H	4	17	15	10	1	1	7	8
	11I	40	28	13	6	5	4	5	14
	11J	21	1	11	13	4	1	9	14
	11K	1	10	26	1	14	8	0	0
	11L	4	12	0	4	0	17	2	6
Control, saline only (N=12)	12A	22	6	1	1	0	7	5	9
	12B	18	3	5	5	9	5	18	12
	12C	6	19	9	4	5	9	25	0
	12D	12	6	3	1	27	1	15	8
	12E	17	7	12	2	20	4	8	0
	12F	3	3	6	1	0	1	15	11
	12G	0	0	0	0	0	1	0	4
	12H	36	26	20	7	3	3	32	2
	12I	6	3	0	12	21	2	35	7
	12J	21	18	6	5	5	2	11	3
	12K	13	5	9	6	7	3	23	2
	12L	13	1	24	2	0	1	2	7

Supplementary Table S10. Barnes maze number of total errors data in the main study.

		Session number (W=week; D=day; '/''=session 1&2)							
	Animal ID	W4D1'	W4D1''	W4D2'	W4D2''	W4D3'	W4D3''	W10D1'	W10D1''
30 µg MC-LR/kg BW/day (N=12)	9A	11	6	8	4	9	3	16	5
	9B	35	32	20	9	12	1	12	3
	9C	11	8	14	2	3	6	10	5
	9D	14	17	20	10	15	8	11	1
	9E	17	14	16	1	18	0	0	2
	9F	19	15	5	4	2	12	10	10
	9G	37	22	6	2	4	5	6	17
	9H	7	4	4	6	1	1	5	14
	9I	14	21	1	16	3	7	3	0
	9J	20	1	14	1	1	3	10	3
	9K	26	5	1	21	9	6	7	6
	9L	35	19	16	7	3	4	9	3
30 mg L-BMAA/kg BW/day (N=12)	10A	28	16	17	11	9	15	9	7
	10B	25	26	22	9	14	7	9	3
	10C	24	6	10	3	1	2	1	0
	10D	23	12	11	11	1	16	30	4
	10E	1	12	8	10	2	5	29	10
	10F	46	36	20	7	3	6	9	16
	10G	19	9	18	4	15	4	17	16
	10H	27	14	9	3	2	10	3	4
	10I	17	13	8		9	0	21	20
	10J	43	8	31	9	8	13	11	9
	10K	24	4	6	8	1	0	2	30
	10L	25	19	17	9	4	7	4	10

30 µg MC-LR +30 mg L-BMAA/kg BW/day (N=12)	11A	19	13	2	8	0	8	11	0
	11B	24	19	5	16	6	1	13	8
	11C	18	8	4	3	0	6	2	0
	11D	20	13	4	5	0	4	0	2
	11E	9	22	3	0	2	3	11	2
	11F	36	18	1	2	0	0	4	8
	11G	18	11	32	7	13	11	20	14
	11H	37	42	15	10	1	1	7	8
	11I	40	28	16	6	5	4	6	14
	11J	33	11	25	13	4	1	10	14
	11K	29	10	26	2	14	8	1	0
	11L	26	12	19	4	0	17	2	6
Control, saline only (N=12)	12A	28	6	1	1	0	7	5	9
	12B	18	13	5	5	9	5	20	12
	12C	46	20	10	4	5	9	25	0
	12D	21	16	3	1	27	1	15	8
	12E	17	19	25	13	20	4	15	0
	12F	3	3	6	1	0	1	15	11
	12G	31	12	0	0	0	1	0	4
	12H	36	27	20	7	3	3	32	2
	12I	21	19	8	12	21	2	35	7
	12J	21	18	6	6	5	2	11	3
	12K	23	10	9	6	7	3	24	2
	12L	13	7	25		1	1	2	7

Supplementary Table S11. Barnes maze mean velocity (cm/s) data in the main study.

		Session number (W=week; D=day; '/''=session 1&2)							
	Animal ID	W4D1'	W4D1''	W4D2'	W4D2''	W4D3'	W4D3''	W10D1'	W10D1''
30 µg MC-LR/kg BW/day (N=12)	9A	1.826	1.948	1.772	6.775	2.202	8.975	5.332	3.040
	9B	5.563	4.907	6.342	7.188	5.363	7.942	9.611	7.201
	9C	3.945	4.164	6.660	6.496	10.695	13.382	7.225	8.186
	9D	3.596	3.461	3.424	1.512	1.883	6.726	8.933	8.311
	9E	2.154	3.085	2.866	7.058	3.781	9.862	5.877	7.147
	9F	3.341	3.637	3.104	4.026	8.495	3.039	2.975	2.550
	9G	5.458	3.102	7.037	5.579	5.202	7.753	5.931	2.401
	9H	1.446	1.613	1.925	6.399	6.426	7.414	6.593	5.463
	9I	2.744	3.204	6.598	-	7.105	5.122	5.294	7.876
	9J	3.633	7.239	4.509	8.168	6.569	7.880	8.345	10.647
	9K	3.422	7.196	3.922	4.070	4.837	2.615	8.553	7.664
	9L	4.522	3.557	7.234	6.674	9.365	10.586	8.330	12.133
30 mg L-BMAA/kg BW/day (N=12)	10A	5.042	3.811	7.876	5.775	7.464	9.493	4.608	6.391
	10B	4.725	4.479	5.454	6.880	5.243	9.232	9.414	8.189
	10C	5.415	9.887	4.956	6.258	6.845	9.468	4.558	7.235
	10D	4.915	4.889	7.773	6.282	6.091	9.160	7.294	11.588
	10E	1.328	2.587	2.231	3.950	5.040	7.653	3.069	1.955
	10F	4.880	4.767	3.388	5.105	8.270	6.250	9.665	4.796
	10G	2.904	7.035	4.960	7.824	7.424	8.257	3.479	6.135

	10H	3.953	2.540	6.225	4.987	7.342	1.827	5.910	4.484
	10I	3.962	3.338	2.322	7.330	3.342	8.944	3.532	2.556
	10J	6.529	8.776	7.712	7.653	7.204	9.078	9.311	8.947
	10K	3.371	2.816	4.021	6.167	6.436	11.298	8.571	6.436
	10L	3.285	3.014	5.537	5.064	6.341	7.348	7.144	7.706
30 µg MC-LR +30 mg L-BMAA/kg BW/day (N=12)	11A	3.913	2.784	9.141	8.704	5.666	7.679	10.027	-
	11B	4.463	4.076	3.620	5.710	7.489	6.199	6.270	9.792
	11C	4.570	4.491	4.351	4.024	4.884	3.572	7.481	6.939
	11D	3.414	2.976	9.888	7.671	4.634	6.192	6.075	7.865
	11E	1.959	2.649	1.689	5.864	7.636	10.123	2.255	5.030
	11F	6.419	7.605	12.397	7.955	5.613	8.148	11.638	5.728
	11G	3.216	2.701	5.526	1.787	3.179	5.710	6.914	7.397
	11H	5.507	5.943	5.508	4.197	6.437	7.495	8.092	8.907
	11I	6.871	5.559	4.576	7.418	7.142	5.923	9.344	9.052
	11J	5.262	7.463	4.695	8.747	7.147	7.232	12.193	8.132
	11K	3.405	3.785	3.986	3.725	5.104	5.278	6.170	10.407
	11L	3.903	3.144	5.951	4.659	9.940	7.052	8.084	9.269
Control, saline only (N=12)	12A	4.866	6.384	10.139	5.988	7.274	5.708	12.220	6.213
	12B	2.859	2.263	2.780	5.432	8.235	7.377	3.033	7.253
	12C	6.883	3.920	7.106	5.952	8.353	8.023	4.542	11.676
	12D	4.290	5.574	7.332	1.794	3.970	7.643	8.613	9.791
	12E	3.271	3.349	3.334	2.422	3.416	2.747	4.499	4.844
	12F	1.421	1.343	2.496	5.463	5.052	6.680	1.580	0.944
	12G	5.370	4.854	6.528	4.373	5.341	6.204	7.454	7.813
	12H	5.813	5.350	5.287	6.974	8.823	9.154	5.654	12.430
	12I	3.560	3.727	3.054	3.271	3.027	3.751	4.457	3.662
	12J	2.759	3.050	4.740	6.709	8.070	4.355	8.257	7.305
	12K	6.059	7.164	7.896	10.800	11.059	12.784	7.987	10.208
	12L	1.945	4.534	4.490	-	5.568	7.762	3.847	6.524

Supplementary Table S12. Barnes maze distance travelled (m) data in the main study.

		Session number (W=week; D=day; '/''=session 1&2)							
	Animal ID	W4D1'	W4D1''	W4D2'	W4D2''	W4D3'	W4D3''	W10D1'	W10D1''
30 µg MC-LR/kg BW/day (N=12)	9A	4.373	4.676	4.254	3.935	5.289	1.228	8.113	3.504
	9B	13.348	11.760	6.560	3.065	4.651	0.559	5.029	0.830
	9C	9.443	6.576	8.562	0.878	1.318	1.370	5.005	2.286
	9D	8.611	8.304	8.218	3.600	4.514	2.427	3.316	0.505
	9E	5.161	7.393	6.879	0.559	6.784	0.513	0.597	1.264
	9F	8.002	5.572	2.257	1.037	1.461	4.800	4.907	2.745
	9G	13.090	7.440	2.455	1.830	1.448	2.872	3.805	5.760
	9H	3.465	2.666	2.102	1.741	0.905	0.652	1.487	3.846
	9I	6.575	7.682	0.623	-	1.188	1.512	0.864	0.460
	9J	8.725	2.038	6.259	0.836	0.836	1.866	3.772	1.082
	9K	8.219	3.713	1.114	7.638	3.661	2.173	3.455	2.428
	9L	10.842	7.379	4.711	1.415	1.491	1.236	2.033	1.437
30 mg L-BMAA/kg BW/day (N=12)	10A	12.077	6.863	6.818	4.481	2.986	3.417	2.245	1.590
	10B	11.336	10.739	8.159	2.900	5.419	1.809	3.871	1.644

	10C	12.970	5.007	3.858	1.056	0.537	0.871	0.755	0.735
	10D	11.796	7.502	4.894	4.699	0.746	4.477	7.738	1.576
	10E	3.186	6.196	4.078	2.929	1.871	2.161	7.382	3.619
	10F	11.689	12.856	5.288	1.515	1.237	1.895	3.101	5.095
	10G	6.963	5.291	7.425	1.959	3.896	1.348	7.888	5.349
	10H	9.478	6.087	4.661	1.225	1.515	2.599	1.007	0.900
	10I	9.506	8.003	4.576	5.119	3.454	0.544	8.468	5.768
	10J	15.676	3.651	10.686	2.627	2.524	2.767	4.313	2.434
	10K	8.088	1.397	3.413	1.663	0.618	0.542	0.816	6.966
	10L	7.876	7.236	4.873	4.140	1.562	2.387	1.755	2.047
30 µg MC-LR +30 mg L-BMAA/kg BW/day (N=12)	11A	9.379	5.859	1.411	3.161	0.585	3.195	4.572	-
	11B	10.697	9.179	2.826	6.272	2.456	1.299	5.172	4.551
	11C	10.262	4.602	2.837	1.233	0.559	1.100	1.161	0.572
	11D	8.185	7.131	2.342	3.050	0.586	1.149	0.530	1.044
	11E	4.696	6.350	1.902	0.488	1.338	1.976	3.319	0.825
	11F	15.410	7.836	1.448	0.910	0.561	0.489	2.160	2.387
	11G	7.720	6.482	13.237	4.284	4.476	4.020	5.714	2.858
	11H	13.211	14.250	5.248	3.758	0.860	0.665	3.418	3.000
	11I	16.490	13.314	6.146	2.267	2.097	1.867	2.130	3.816
	11J	12.609	5.845	9.072	3.261	1.584	0.775	3.794	4.177
	11K	8.159	5.320	9.538	1.165	4.181	2.909	0.637	0.491
	11L	9.354	5.455	5.156	1.170	1.121	4.468	1.242	2.603
Control, saline only (N=12)	12A	11.671	3.110	1.136	0.838	0.809	1.448	2.679	3.092
	12B	6.862	4.512	2.277	2.181	2.102	1.287	6.258	2.449
	12C	16.508	8.913	4.065	1.562	1.958	4.358	8.546	0.607
	12D	10.281	8.027	1.859	1.402	7.917	0.599	4.603	3.846
	12E	7.844	8.037	8.004	5.815	8.202	1.303	5.395	0.562
	12F	15.410	7.836	1.448	0.910	0.561	0.489	2.160	2.387
	12G	12.871	4.710	0.585	0.588	0.538	0.526	0.572	1.400
	12H	13.932	11.076	6.116	2.951	1.320	1.326	8.527	1.134
	12I	8.530	8.931	6.289	6.348	7.254	1.119	10.682	1.854
	12J	6.626	6.022	1.407	1.363	1.246	1.003	3.560	1.087
	12K	14.542	6.230	2.242	1.728	4.565	1.493	8.313	1.800
	12L	4.665	4.588	7.274	-	0.584	0.528	1.117	2.250

Supplementary Table S13. Main study OF data. Week 8, day 1.

	Animal ID	Total distance traveled (m)	Mean velocity (cm/s)	Cumulative duration (%) in center zone
30 µg MC-LR/kg BW/day (N=12)	9A	48.97	8.168	18.87
	9B	34.44	5.744	23.28
	9C	24.92	4.156	33.17
	9D	39.82	6.641	8.42
	9E	21.54	3.593	25.62
	9F	25.20	4.203	11.63
	9G	34.23	5.708	9.83
	9H	29.86	4.978	14.56
	9I	45.12	7.524	24.88
	9J	37.90	6.319	18.24

	9K	24.30	4.054	14.21
	9L	37.07	6.183	18.11
30 mg L-BMAA/kg BW/day (N=12)	10A	25.65	4.278	24.68
	10B	27.34	4.561	48.42
	10C	25.32	4.222	22.23
	10D	50.23	8.378	21.40
	10E	41.23	6.877	17.09
	10F	35.35	5.896	24.51
	10G	34.19	5.702	19.02
	10H	45.68	7.617	20.82
	10I	41.52	6.924	14.50
	10J	42.84	7.143	16.10
	10K	27.36	4.565	28.83
	10L	38.55	6.428	21.41
30 µg MC-LR +30 mg L-BMAA/kg BW/day (N=12)	11A	31.66	5.283	13.99
	11B	32.58	5.439	11.90
	11C	24.24	4.043	16.12
	11D	26.87	4.482	39.14
	11E	25.26	4.213	14.29
	11F	25.84	4.310	31.53
	11G	44.63	7.441	34.57
	11H	60.88	10.150	11.81
	11I	39.04	6.509	17.35
	11J	38.87	6.481	7.85
	11K	53.79	8.972	8.92
	11L	31.71	5.289	5.96
Control, saline only (N=12)	12A	29.86	4.980	19.66
	12B	25.81	4.307	12.12
	12C	41.34	6.899	21.41
	12D	38.70	6.459	38.28
	12E	36.09	6.020	0.84
	12F	20.58	3.432	47.27
	12G	50.62	8.440	25.54
	12H	35.76	5.962	14.24
	12I	45.22	7.540	23.09
	12J	47.59	7.935	20.51
	12K	32.36	5.398	11.17
	12L	22.48	3.750	23.80

Supplementary Table S14. Main study NLR data. Week 8, day 2, session 2 (session 1 was for acclimatization without using video recording).

	Animal ID	Number of nose-point entries Zone 1	Zone 2	Z1+Z2	RATIO fZ2/fZ1	Discrimination index	Cumulative nose-point duration (s) in zone facing object Zone 1	Zone 2	Z1+Z2	RATIO cZ2/cZ1	Discrimination index
30 µg MC-LR/kg BW/day (N=12)	9A	6	9	15	1.500	0.600	5.026	7.539	12.565	1.500	0.600
	9B	6	12	18	2.000	0.667	3.696	6.652	10.347	1.800	0.643
	9C	3	9	12	3.000	0.750	1.478	6.208	7.687	4.200	0.808
	9D	5	5	10	1.000	0.500	1.922	2.217	4.139	1.154	0.536
	9E	6	7	13	1.167	0.538	3.696	4.139	7.834	1.120	0.528
	9F	6	4	10	0.667	0.400	2.809	2.661	5.469	0.947	0.486
	9G	3	5	8	1.667	0.625	1.973	3.794	5.766	1.923	0.658
	9H	4	3	7	0.750	0.429	2.124	2.731	4.856	1.286	0.563
	9I	9	16	25	1.778	0.640	9.256	17.602	26.859	1.902	0.655
	9J	9	12	21	1.333	0.571	7.284	12.898	20.182	1.771	0.639
	9K	5	5	10	1.000	0.500	6.070	6.373	12.443	1.050	0.512
	9L	5	4	9	0.800	0.444	4.552	2.428	6.980	0.533	0.348
30 mg L-BMAA/kg BW/day (N=12)	10A	4	4	8	1.000	0.500	3.400	2.365	5.765	0.696	0.410
	10B	7	8	15	1.143	0.533	6.061	5.913	11.973	0.976	0.494
	10C	10	7	17	0.700	0.412	12.417	7.834	20.251	0.631	0.387
	10D	11	10	21	0.909	0.476	6.652	5.765	12.417	0.867	0.464
	10E	4	4	8	1.000	0.500	3.548	4.139	7.687	1.167	0.538
	10F	6	5	11	0.833	0.455	5.026	2.956	7.982	0.588	0.370
	10G	12	10	22	0.833	0.455	14.112	6.980	21.093	0.495	0.331
	10H	3	5	8	1.667	0.625	3.338	4.401	7.739	1.318	0.569
	10I	9	16	25	1.778	0.640	10.774	16.237	27.011	1.507	0.601
	10J	9	7	16	0.778	0.438	9.408	8.649	18.058	0.919	0.479
	10K	5	5	10	1.000	0.500	4.552	4.249	8.801	0.933	0.483
	10L	6	5	11	0.833	0.455	4.552	7.132	11.684	1.567	0.610
30 µg MC-LR +30 mg L-BMAA/kg BW/day (N=12)	11A	5	4	9	0.800	0.444	2.661	4.139	6.800	1.556	0.609
	11B	3	6	9	2.000	0.667	2.069	4.878	6.948	2.357	0.702
	11C	6	8	14	1.333	0.571	4.435	7.095	11.530	1.600	0.615

	11D	8	3	11	0.375	0.273	9.017	2.217	11.234	0.246	0.197
	11E	5	5	10	1.000	0.500	5.174	4.730	9.904	0.914	0.478
	11F	18	16	34	0.889	0.471	11.087	12.269	23.356	1.107	0.525
	11G	7	10	17	1.429	0.588	8.346	14.264	22.610	1.709	0.631
	11H	9	11	20	1.222	0.550	7.891	8.649	16.540	1.096	0.523
	11I	4	7	11	1.750	0.636	2.124	6.829	8.953	3.214	0.763
	11J	2	3	5	1.500	0.600	1.517	3.338	4.856	2.200	0.688
	11K	5	6	11	1.200	0.545	5.615	4.401	10.015	0.784	0.439
	11L	4	5	9	1.250	0.556	3.035	4.097	7.132	1.350	0.574
Control, saline only (N=12)	12A	3	7	10	2.333	0.700	1.626	5.174	6.800	3.182	0.761
	12B	3	4	7	1.333	0.571	2.217	2.956	5.174	1.333	0.571
	12C	9	6	15	0.667	0.400	6.504	4.435	10.939	0.682	0.405
	12D	4	6	10	1.500	0.600	1.330	8.426	9.756	6.333	0.864
	12E	3	5	8	1.667	0.625	3.400	4.287	7.687	1.261	0.558
	12F	4	1	5	0.250	0.200	2.513	1.035	3.548	0.412	0.292
	12G	7	5	12	0.714	0.417	7.284	2.580	9.863	0.354	0.262
	12H	5	11	16	2.200	0.688	3.642	13.050	16.692	3.583	0.782
	12I	9	4	13	0.444	0.308	15.023	3.945	18.968	0.263	0.208
	12J	5	10	15	2.000	0.667	4.856	13.050	17.906	2.688	0.729
	12K	7	5	12	0.714	0.417	5.766	2.731	8.498	0.474	0.321
	12L	2	1	3	0.500	0.333	1.669	0.910	2.580	0.545	0.353

Supplementary Table S15. Main study NOR data. Week 8, day 2, session 3.

	Animal ID	Number of nose-point entries Zone 1	Zone 2	Z1+Z2	RATIO fZ2/fZ1	Discrimination index	Cumulative nose-point duration (s) in zone facing object Zone 1	Zone 2	Z1+Z2	RATIO cZ2/cZ1	Discrimination index	
30 µg MC-LR/kg BW/day (N=12)	9A	5	7	12	1.400	0.583	3.400	7.243	10.643	2.130	0.681	
	9B	9	12	21	1.333	0.571	4.287	10.939	15.225	2.552	0.718	
	9C	1	6	7	6.000	0.857	1.330	7.835	9.165	5.889	0.855	
	9D	1	2	3	2.000	0.667	0.591	0.887	1.478	1.500	0.600	
	9E	6	2	8	0.333	0.250	2.661	2.069	4.730	0.778	0.438	
	9F	5	8	13	1.600	0.615	2.956	6.652	9.608	2.250	0.692	
	9G	3	3	6	1.000	0.500	3.945	3.945	7.891	1.000	0.500	
	9H	-	-	-	-	-	-	-	-	-	-	experimental error
	9I	15	8	23	0.533	0.348	24.279	12.443	36.722	0.513	0.339	
	9J	8	10	18	1.250	0.556	14.871	16.389	31.260	1.102	0.524	
	9K	3	2	5	0.667	0.400	5.159	3.187	8.346	0.618	0.382	
	9L	7	3	10	0.429	0.300	4.401	5.311	9.712	1.207	0.547	
30 mg L-BMAA/kg BW/day (N=12)	10A	5	8	13	1.600	0.615	2.661	6.208	8.869	2.333	0.700	
	10B	-	-	-	-	-	-	-	-	-	-	experimental error
	10C	4	3	7	0.750	0.429	2.956	2.956	5.913	1.000	0.500	
	10D	12	11	23	0.917	0.478	6.800	12.713	19.512	1.870	0.652	
	10E	1	1	2	1.000	0.500	0.443	0.739	1.183	1.667	0.625	
	10F	2	4	6	2.000	0.667	1.183	2.956	4.139	2.500	0.714	
	10G	4	3	7	0.750	0.429	4.401	1.821	6.222	0.414	0.293	
	10H	2	2	4	1.000	0.500	1.973	3.338	5.311	1.692	0.629	
	10I	9	14	23	1.556	0.609	11.684	14.719	26.404	1.260	0.557	
	10J	14	6	20	0.429	0.300	23.521	5.008	28.528	0.213	0.176	
	10K	4	2	6	0.500	0.333	6.222	1.973	8.194	0.317	0.241	
	10L	8	10	18	1.250	0.556	14.264	10.015	24.279	0.702	0.413	
30 µg MC-LR +30 mg L-BMAA/kg BW/day (N=12)	11A	7	7	14	1.000	0.500	3.400	4.730	8.130	1.391	0.582	
	11B	5	11	16	2.200	0.688	4.139	19.217	23.356	4.643	0.823	
	11C	5	3	8	0.600	0.375	5.322	3.991	9.313	0.750	0.429	

	11D	3	5	8	1.667	0.625	1.330	4.878	6.208	3.667	0.786	
	11E	4	3	7	0.750	0.429	3.696	6.061	9.756	1.640	0.621	
	11F	17	7	24	0.412	0.292	12.121	6.061	18.182	0.500	0.333	
	11G	5	3	8	0.600	0.375	10.319	5.766	16.085	0.559	0.358	
	11H	14	3	17	0.214	0.176	27.314	2.883	30.197	0.106	0.095	
	11I	13	9	22	0.692	0.409	20.334	15.630	35.964	0.769	0.435	
	11J	4	5	9	1.250	0.556	4.249	7.132	11.381	1.679	0.627	
	11K	5	4	9	0.800	0.444	3.945	6.525	10.470	1.654	0.623	
	11L	5	6	11	1.200	0.545	4.856	7.132	11.988	1.469	0.595	
Control, saline only (N=12)	12A	3	4	7	1.333	0.571	1.774	4.139	5.913	2.333	0.700	
	12B	-	-	-	-	-	-	-	-	-	-	experimental error
	12C	6	6	12	1.000	0.500	4.878	4.878	9.756	1.000	0.500	
	12D	10	5	15	0.500	0.333	9.460	5.469	14.930	0.578	0.366	
	12E	3	3	6	1.000	0.500	2.809	0.887	3.696	0.316	0.240	
	12F	9	5	14	0.556	0.357	4.582	2.069	6.652	0.452	0.311	
	12G	2	1	3	0.500	0.333	2.276	1.214	3.490	0.533	0.348	
	12H	5	6	11	1.200	0.545	9.560	10.926	20.486	1.143	0.533	
	12I	12	6	18	0.500	0.333	26.555	6.525	33.080	0.246	0.197	
	12J	7	8	15	1.143	0.533	6.677	7.891	14.568	1.182	0.542	
	12K	5	5	10	1.000	0.500	5.008	5.159	10.167	1.030	0.507	
	12L	7	7	14	1.000	0.500	12.595	9.256	21.851	0.735	0.424	

Supplementary Table S16. Barnes maze startdirection error (0 to 180 degrees) data from the scopolamine study.

Animal ID	Treatment	D1'	D1''	D2'	D2''	D3'	D3''
		Session number (D=day; '/''=session 1&2)					
2	saline	162	162	18	108	18	162
3	scopolamine	108	144	90	72	108	72
4	saline	18	-	72	54	126	54
5	scopolamine	54	90	54	36	54	72
6	saline	18	54	144	18	0	54
7	scopolamine	108	-	72	54	36	126
8	saline	162	144	162	18	36	162
9	scopolamine	108	90	126	18	90	54
10	saline	18	144	18	18	0	18
11	scopolamine	90	162	162	36	90	54
12	saline	18	54	90	54	18	18
13	scopolamine	108	108	126	54	162	126
14	saline	36	90	54	162	18	54
15	scopolamine	108	54	108	18	90	108

Supplementary Table S17. Barnes maze primary latency (s) data from the scopolamine study.

Animal ID	Treatment	D1'	D1''	D2'	D2''	D3'	D3''
		Session number (D=day; '/''=session 1&2)					
2	saline	26.34	136.32	199.32	122.08	81.76	21.68
3	scopolamine	64.88	35.56	91.44	192.48	131.84	16.88
4	saline	21.84	25.16	121.84	137.28	115.28	21.76
5	scopolamine	37.92	82.96	180.64	119.60	95.60	-
6	saline	3.00	-	13.20	77.92	16.16	40.08
7	scopolamine	90.88	62.80	97.84	240	240	240
8	saline	212.80	23.20	15.04	6.64	54.96	21.76
9	scopolamine	240	187.45	-	12.50	9.76	6.24
10	saline	51.76	217.04	46.80	48.08	1.68	123.52
11	scopolamine	27.44	240	96.48	162.32	41.36	135.12
12	saline	121.52	240	20.80	7.52	9.12	8.56
13	scopolamine	240	198.88	198.64	132.32	226.96	31.92
14	saline	72.72	240	48.16	186.64	41.20	52.32
15	scopolamine	105.12	19.52	172.64	10.96	240	210.00

Supplementary Table S18. Barnes maze escape latency (s) data from the scopolamine study.

Animal ID	Treatment	D1'	D1''	D2'	D2''	D3'	D3''
		Session number (D=day; '/''=session 1&2)					
2	saline	240	240	240	240	96.16	28.16
3	scopolamine	240	240	240	240	240	240
4	saline	240	72.52	135.36	141.60	119.28	26.96
5	scopolamine	240	240	240	240	240	-

6	saline	45.88	-	37.60	81.20	19.20	42.64
7	scopolamine	240	240	240	240	240	240
8	saline	240	30.72	67.04	9.76	57.12	23.92
9	scopolamine	240	240	-	240	240	-
10	saline	240	240	240	58.40	3.40	126.00
11	scopolamine	240	240	240	240	240	240
12	saline	240	240	79.52	57.52	13.68	12.80
13	scopolamine	240	240	218.40	152.80	240	240
14	saline	240	240	74.00	194.80	45.36	59.04
15	scopolamine	240	240	240	69.44	240	221.84

Supplementary Table S19. Barnes maze number of primary errors data from the scopolamine study.

		Session number (D=day; '/''=session 1&2)					
Animal ID	Treatment	D1'	D1''	D2'	D2''	D3'	D3''
2	saline	6	8	22	14	5	7
3	scopolamine	13	5	19	35	4	4
4	saline	5	-	15	19	16	5
5	scopolamine	8	16	21	22	35	15
6	saline	1	-	2	9	0	6
7	scopolamine	16	-	10	20	11	4
8	saline	39	3	4	1	12	5
9	scopolamine	24	30	-	3	3	3
10	saline	7	15	9	1	0	10
11	scopolamine	4	29	12	16	6	29
12	saline	16	24	5	3	1	1
13	scopolamine	28	24	17	23	37	8
14	saline	11	14	3	14	3	2
15	scopolamine	13	3	24	2	5	12

Supplementary Table S20. Barnes maze number of total errors data from the scopolamine study.

		Session number (D=day; '/''=session 1&2)					
Animal ID	Treatment	D1'	D1''	D2'	D2''	D3'	D3''
2	saline	25	22	23	21	5	7
3	scopolamine	42	-	50	41	13	22
4	saline	47	-	15	19	16	5
5	scopolamine	41	33	39	52	106	15
6	saline	14	-	2	9	0	6
7	scopolamine	38	-	23	20	11	4
8	saline	43	3	15	1	12	5
9	scopolamine	24	38	-	55	41	-
10	saline	29	15	31	1	0	10
11	scopolamine	30	29	25	26	30	41
12	saline	32	24	17	7	1	1
13	scopolamine	28	24	17	23	37	55
14	saline	26	14	3	14	3	2

15	scopolamine	23	27	24	2	5	12

Supplementary Table S21. Barnes maze mean velocity (cm/s) data from the scopolamine study.

		Session number (D=day; '/''=session 1&2)					
Animal ID	Treatment	D1'	D1''	D2'	D2''	D3'	D3''
2	saline	4.463	3.910	4.516	2.604	2.663	5.675
3	scopolamine	6.140	-	6.836	5.137	2.477	2.558
4	saline	7.113	3.540	4.762	6.839	5.694	4.352
5	scopolamine	7.352	5.622	5.323	6.578	8.570	2.785
6	saline	7.990	-	4.703	6.520	-	6.173
7	scopolamine	9.065	5.184	2.813	3.245	1.343	0.779
8	saline	6.493	6.049	7.914	8.314	6.984	7.582
9	scopolamine	9.920	7.234	-	6.915	6.392	-
10	saline	4.989	3.721	4.806	1.277	15.692	3.198
11	scopolamine	4.984	3.592	5.862	5.580	4.728	4.444
12	saline	5.484	4.092	6.486	5.314	6.098	5.689
13	scopolamine	6.810	5.654	4.046	6.877	5.225	9.543
14	saline	6.030	3.212	2.975	4.083	3.290	2.629
15	scopolamine	6.157	3.918	3.055	2.258	1.461	2.619

Supplementary Table S22. Barnes maze distance travelled (m) data from the scopolamine study.

		Session number (D=day; '/''=session 1&2)					
Animal ID	Treatment	D1'	D1''	D2'	D2''	D3'	D3''
2	saline	10.712	9.384	10.839	6.251	2.501	1.593
3	scopolamine	14.735	-	16.405	12.329	5.945	6.140
4	saline	17.072	-	6.446	9.684	6.782	1.170
5	scopolamine	17.645	13.493	12.774	15.788	20.568	-
6	saline	3.560	-	1.765	5.289	-	2.632
7	scopolamine	21.755	-	6.752	7.789	3.224	1.871
8	saline	15.584	1.844	5.299	0.811	3.984	1.807
9	scopolamine	23.807	17.362	-	16.596	15.341	-
10	saline	11.974	8.929	11.534	0.744	0.477	4.030
11	scopolamine	11.961	8.620	14.068	13.393	11.348	10.217
12	saline	13.160	9.820	5.158	3.052	0.829	0.728
13	scopolamine	16.345	13.569	8.807	10.492	12.539	22.902
14	saline	14.472	7.710	2.185	7.915	1.482	1.552
15	scopolamine	14.777	9.404	7.332	1.559	3.507	5.804
